# Design Requirements for Cardiac Telerehabilitation Technologies Supporting Athlete Values: Qualitative Interview Study

**DOI:** 10.2196/62986

**Published:** 2025-04-17

**Authors:** Irina Bianca Șerban, Lonneke Fruytier, Steven Houben, Sara Colombo, Danny van de Sande, Hareld Kemps, Aarnout Brombacher

**Affiliations:** 1 Faculty of Industrial Design Eindhoven University of Technology Eindhoven The Netherlands; 2 Department of Cardiology Máxima Medical Center Veldhoven The Netherlands; 3 Faculty of Industrial Design Engineering Delft University of Technology Delft The Netherlands

**Keywords:** athletes with established coronary artery disease, cardiac telerehabilitation, value-sensitive design, sports monitoring, card sorting, qualitative research, artificial intelligence, AI

## Abstract

**Background:**

Cardiac telerehabilitation (CTR) interventions can provide accessible and affordable remote rehabilitation services. However, as cardiac rehabilitation (CR) primarily targets inactive patients, little is known about the experiences with CR of highly active patients (ie, recreational athletes or, simply, athletes) with established coronary artery disease. Consequently, existing CTR interventions do not address the specific needs of the athletic subpopulation. Understanding the needs and values of athletes is crucial for designing meaningful CTR interventions that enhance user acceptance and engagement, thereby facilitating effective rehabilitation for this patient subgroup.

**Objective:**

This study aimed to inform the design of technologies that facilitate CTR for athletes. We intended to identify athletes’ values related to CR, including health and sports tracking, as well as high-level requirements for technologies that can facilitate the CTR of athletes according to the identified values.

**Methods:**

We used value-sensitive design with a human-centric design approach to elicit design requirements for CTR that can serve athletes with established coronary artery disease. To identify athletes’ values, we conducted 25 value-oriented semistructured interviews with 15 athletic patients and 10 health care professionals involved in CR programs. In a second phase, we conducted 6 card-sorting focus group sessions with 13 patients and 7 health care professionals to identify desired CTR features. Finally, we derived high-level CTR technology requirements connected to the athletes’ needs and values.

**Results:**

We defined 12 athlete values divided into 3 categories: *body centric*, *care centric*, and *data and technology centric*. We clustered findings from the card-sorting activity into CTR technology requirements, such as *remotely monitored sport-specific training* and *training data representations next to clinical limitations*, and paired them with corresponding values.

**Conclusions:**

Athletes have distinct values and health goals in CR compared to general populations targeted by CTR interventions. Designing patient-centric CTR interventions that address these needs is crucial to support optimal recovery, safe return to sports, and adherence to CTR technologies in the home environment.

## Introduction

### Study Background: Cardiac Rehabilitation, Cardiac Telerehabilitation, and the Athletic Cardiac Subpopulation

Cardiovascular disease (CVD) remains one of Europe’s leading causes of mortality [1]. Interventions focused on promoting a healthy lifestyle, such as exercise-based cardiac rehabilitation (CR), can have a significant impact on the outlook of individuals with CVDs, reducing morbidity and mortality and increasing their quality of life [2,3]. In recent years, especially after the COVID-19 pandemic, the acceptance of cardiac telerehabilitation (CTR)—or virtual CR—has been on the rise in many jurisdictions [4,5]. CTR mostly relies on digital and wearable technologies that facilitate delivering CR services to patients in their own homes, encompassing elements such as video-streamed exercise and therapy sessions and remote monitoring of health and behavior, as well as applications enabling patient–health care professional (HCP) communication and information provision [6-8]. For positive rehabilitation outcomes to be achieved, users (ie, patients) need to accept, engage with, and adopt CTR interventions—this requires the technology to be usable and offer perceptible value for the patients [9,10].

To better recognize what drives the use and perceived value of technology-centric CTR, recent human-computer interaction (HCI) literature has underlined the importance of using human-centric design in CTR development by acknowledging the collective perspectives of the *prospective users*, including their experiences and needs [4,11]. In this study, we looked into the experiences and needs of an underresearched prospective CTR user group—*athletic patients with established coronary artery disease* *(CAD)*, the most prevalent cardiovascular condition [12]. Although physical activity is associated with a significantly lower risk of CVDs, highly active individuals are not immune to the development of CAD, and vigorous exercise can be the trigger for life-threatening cardiac events [13,14]—approximately 10.6% of all acute myocardial infarctions may be attributed to physical exertion [15]. Middle-aged and older athletes are at the highest risk of adverse cardiovascular events during exercise as the prevalence of CAD increases with age [15]. Although the prevalence of athlete patients in CR programs is not well defined, there has been growing attention toward middle-aged and older athletic patients with cardiac conditions in the past 10 years—more and more studies show that athletes can develop CAD and its consequences [16], investigating the significant impact of adverse events on sports practice and discussing the exercise recommendations and restrictions that apply based on the type and severity of the underlying problem [2]. To foreground this type of CTR user, we adopt the term *athlete* from clinical literature as it is the most accurate description of our target group—highly active patients who engage in at least 4 hours per week of exercise and potentially participate in competitive athletics (eg, cycling, triathlon, and marathon) recreationally [17].

### Research Gap and Opportunity: Designing CTR for Athletes

There is a notable scarcity of research on athletes in CR, especially on technological interventions for athletes in CR, with recent work on this subgroup focusing mainly on clinical evidence (eg, etiology and prevention strategies [16]). This lack of knowledge can be attributed to the fact that, in a largely sedentary population, the central objective of rehabilitation is *improving and increasing* physical activity [2]. Consequently, the primary focus of most existing health technology, including CTR interventions, has been providing support to predominantly inactive and frail populations in their effort to combat sedentary lifestyles and instill healthy lifestyle habits [18-22]. Therefore, we highlight the lack of adequate consideration for the perspectives of athletic patient populations who are already physically active and require guidance on safely and gradually resuming sports rather than being educated on the importance of physical activity in the design and development of technologies that support CTR.

Despite the incomplete evidence on athletes’ rehabilitation needs and challenges, there is evidence suggesting that athletes with established CAD may perceive traditional CR programs as ill-fitting due to disparities in exercise pace and objectives [23]. They require personalized guidance to ensure optimal recovery, mitigate the risk of exercise-induced events, and safely resume their preferred sports practice [24]. Current CTR technologies designed using behavior change and persuasive techniques to motivate general patient populations to adopt an active lifestyle [25-27] might not serve the objectives of the athletic patient population [23]. At the intersection of HCI and clinical research, there is an increasing focus on the significance of tailoring cardiac technologies to align with precision medicine principles. This involves *considering individual patient differences*, ultimately aiming to mitigate complications associated with CVDs [28,29]. Recent efforts from the HCI community have looked into tailoring CTR content and mode of delivery to various user groups (eg, by identifying the right CTR interfaces based on older patients’ technology familiarity or by customizing tracking devices based on lifestyle [4,30,31]).

Failing to acknowledge the nuanced needs of the athletic user group in the design of CTR interventions can result in poor user experience and low technology engagement and adoption. This, in turn, can affect the effectiveness of the rehabilitation treatment and induce serious psychological side effects such as depression and anxiety [4,32-34].

### Study Objectives

Our overall aim was to provide insights into the design of CTR technologies aiming to support athletic patients. Our study used a human-centric design approach called value-sensitive design (VSD)—a theory-driven approach to technology design that integrates human values throughout the design process [32,35], with values reflecting what individuals consider important in life and care, driving their needs (in this work, the terms *needs* and *values* are to be regarded in the context of the VSD framework) [36].

To achieve our aim, we had the following objectives: (1) to elicit athletes’ values regarding CR, including health and behavior tracking; and (2) to define design requirements for CTR technologies that meet the athletes’ values.

We conducted 25 individual, value-oriented semistructured interviews with 15 athletes with established CAD and 10 HCPs involved in CR programs to understand athletes’ current challenges in CR, behavior tracking, and technology-centric care and derive values. To identify technology-focused CTR design requirements that can cater to these needs and values, we conducted 6 card-sorting focus group (FG) sessions with 13 patients and 7 HCPs.

Our work contributes in three key ways: (1) we inform the design and development of CTR technologies personalized for the underresearched athletic subgroup of patients with cardiac conditions; (2) we uncover the currently underrepresented needs, values, and desired CTR features of athletes with cardiac conditions; and (3) we use VSD as an investigation method for participatory design, something novel in the current design of CTR [37].

### Related Work

The research presented in this paper spans multiple domains, encompassing clinical research on athletes with heart conditions, characteristics of recreational athletes, the design of CTR interventions, and human-centered approaches to CTR and health technology (ie, eHealth) design overall.

#### Recreational Athletes in Cardiac and HCI Research

Existing studies on the requirements and perceptions of CTR technologies have been conducted in heterogeneous populations and in patients with low exercise capacity [38,39] and a sedentary lifestyle [40] or specifically in older adults [41]. Nonetheless, the needs of highly active patients (ie, athletes) regarding CTR have not yet been addressed. In a wider perspective, apart from a few clinical case reports, there is a clear lack of documented information regarding athletes in CR [23,42,43]. Existing knowledge of this population points to the many benefits that CTR interventions could have for their rehabilitation. First, athletes need tailored evaluation and sport-specific guidance before resuming intense activities, preferably in their sports setting and not in traditional hospital-based CR [24]. Furthermore, there is evidence that athletes are eager to return to sports quickly [23,42-44]. CTR helps achieve this by (1) starting right after hospitalization, shortening the outpatient gap between hospitalization and the CR program; and (2) providing remote supervision to alleviate sport-related anxiety in the posthospitalization phase [23,45]. In addition, in the realm of HCI literature concerning recreational athletes, there is a notable emphasis on their heightened data literacy and the practice of monitoring health and performance via wearable and mobile technologies [46]. Athletes use fitness trackers and mobile apps to measure palpable goals such as speed, duration, and intensity and recalibrate their subjective, lived sense of performance [47]. Digital and data literacy and self-monitoring proficiency are positive indicators for adherence to CTR and more effective self-management [45,48].

Given that our research sits at the crossroads of recreational athletes and patients with cardiac conditions, we delved deeper into the established traits of these groups. Regarding recreational athletes, Tholander and Nylander [47] highlight the significance of soft, less measurable goals such as lifestyle and well-being maintenance and identity building as a foundation for sports performance and exercise. Dionigi et al [49] reinforce the intrinsic motivation and self-reliance that athletes exhibit, with less dependence on social support or external incentives. On the other hand, the values of patients with cardiac conditions regarding health care have been identified in recent work by Bente et al [50]. These values include the need for security; support; not wanting to feel anxious; tailoring of treatment; and personalized, accessible care. Nonetheless, the convergence of values and traits between these 2 groups remains unexplored. Transitioning from a highly active lifestyle to a sudden halt caused by a CAD diagnosis can alter one’s relationship with sports, health, and technology. We aimed to investigate this aspect in our research.

#### CTR: Features and Current Designs

During CR, patients are offered hospital-based support that consists of therapies addressing healthy and sustainable behaviors (eg, group exercise training 2-3 times per week, education, psychoeducational therapy, and smoking cessation therapy) with a focus on increasing physical activity and fitness [51,52]. CTR interventions facilitate the delivery of these therapies in the home environment [34]. CTR interventions can be remote (with asynchronous communication between clinicians and patients during exercise sessions), virtual (with real-time audiovisual communication between clinicians and patients during exercise sessions), or hybrid (a combination of in-person CR and remote or virtual services) [33]. While our study emphasized technology-driven rehabilitation design, we did not exclude elements of hybrid interventions (eg, face-to-face consultations) from our card-sorting FG. Previous research indicates that certain patients prefer a blend of remote and in-person services for the social benefits [11]. Thus, we aimed to provide all options to our participants without exclusion.

According to recent reviews of CTR design features and the definition of CTR, there are four clusters of design features: (1) *education and assistance*; (2) *consultations, coaching, and guidance*; (3) *monitoring behaviors for supervision and self-management*; and (4) *peer support* [4,53,54]. Nonetheless, in recent work, Andersen et al [45] point out that existing cardiac technologies mostly focus only on individual information processing. Within our work, we aimed to address the relational aspects of rehabilitation in the home by renaming the fourth category as *coexperience (social aspect)*, focusing on features related to data in a social context and connecting with peers and family.

CTR features encompass technology and care service features. Technology features facilitate users’ achievement of a certain goal (eg, data interpretation and irregularity alerts), whereas care service features involve patient actions supported by technology (eg, consultation requests) and services for CTR technology provision (eg, wearable technology support) [4]. These 2 categories are strongly intertwined as the accuracy and engagement of the technology can influence the quality of the care service (eg, asynchronous SMS text messaging or video calling facilitating consultations [55,56], wearable sensors enabling remote supervision [30], or gamified user experiences providing exercise instructions [57]), whereas the delivered care service can dictate the perceived value and engagement with technology (eg, the type of information provided through digital technologies [58] or allowing family members to participate in remote exercise sessions [59]). HCI practitioners design or optimize interactions between care technologies and patients based on the nature of the intervention (remote, virtual, or hybrid) and its focus (eg, educational content technologies). For instance, in a recent study, Tadas et al [11] looked into patient-data interaction mechanisms that facilitate the transition from program to self-management. Similarly, Kjærup et al [60] investigated user preferences for a data collection application that turns patients into active diagnostic agents. On the other hand, by tackling ways in which care services are provided through technologies, Sankaran et al [27] designed more intelligible CTR exercise prescriptions and progress reports to stimulate patient behavior change motivation in mixed sedentary and more active patients. However, generally, the aim of current CTR interventions revolves around supporting patients in learning about and maintaining healthy behaviors by using various techniques, theories, or methods related to cognition and behavior in their design [37,54,61]. A multitude of CTR interventions are designed using behavior change techniques [55,58,62] and motivational techniques [59,63,64] to stimulate patients to implement healthier behaviors and sustain them in self-management [8,37]. For instance, Dithmer et al [65] redesigned remote training sessions into a collaborative game by using collaboration, goals, and rewards in an attempt to increase adherence to the physical activity component of CTR. On the other hand, Ding et al [66] used individualized goal-setting strategies based on exercise progress to engage patients with myocardial infarction in home-based exercise, whereas Hallberg et al [67] used motivational messages and periodic clinical feedback to support patients in hypertension self-management. Nonetheless, these interventions might not align well with existing indications of operational preferences of the athletic population regarding lifestyle choices, potentially leading to low end-user engagement, satisfaction, and self-management [23,32]. In this study, we aimed to advance the design of technology-centric, exercise-based CTR interventions by investigating the larger spectrum of CTR design features through the lens of the values of athletes with cardiac conditions.

#### Applying Human-Centered Design Through Human Values

Although CTR has been proven a safe and effective alternative to center-based CR for patients with CAD [39,54], engagement with and adherence to the program are highly impacted by the usability, reliability, and engagement of the designed intervention [6,68]. Ensuring ease of use and user satisfaction can be achieved through human-centered design (HCD) approaches (eg, user research or participatory design)—increasingly used in eHealth design [69]. Recent HCI research on eHealth for chronic conditions stresses the need for human-centric, in-depth investigations into target group challenges. For instance, Min et al [70] investigated epilepsy care challenges and proposed design guidelines for information management and care coordination technology. Cha et al [71] studied parental risk management for the glucose levels of children with diabetes and suggested support technology guidelines. Similarly, Sepehri et al [72] consulted parents of children with health complexity to gather needs for digital management systems. Finally, Bhat et al [73] gathered qualitative data on informal caregivers’ roles in chronic disease management to inform technologically assisted care options. In the context of CTR interventions, several studies use human-centered methods to elicit needs or desired functionalities. For instance, the Teledialog CTR program was designed through qualitative user need elicitation, cultural probes, and user testing [74]. Similarly, Beleigoli et al [31] conceptualized a technology-centric CTR program through multi-stakeholder engagement in barrier elicitation and co-design. By innovatively using data-enabled design mixed with human-centric methods, Khanshan et al [75] iterated on a CTR platform. Using a more traditionally qualitative approach, Dinesen et al [76] investigated the needs of homogeneous populations regarding CR and, accordingly, designed a system of multimodal CTR technologies. Nonetheless, a recent systematic scoping review points out that less than one-fifth of CTR interventions reported including end users in the design and development of these systems [54]. HCI practitioners and technology designers are increasingly encouraged to actively involve patients in the design and development of CTR technologies [6,8].

In our pursuit to define design guidelines for CTR interventions that address the needs of the athlete subpopulation, we conducted an empirical study inspired by the VSD tripartite methodology [77,78]. Holistic approaches to eHealth design, such as the Centre for eHealth Research Roadmap, stress the importance of identifying the diverse and potentially conflicting values of various stakeholders (eg, “what do patients value in health and life that they ultimately expect technology to cater to?” [79]). There are recent HCI inquiries that demonstrate the application of VSD methods in the design and development of eHealth interventions (eg, an artificial intelligence application targeting individuals with dementia [80] or a mobile app aimed at weight loss maintenance [81]). An in-depth inquiry into the design of cardiac technologies by Cruz-Martínez et al [32] underlines the importance of addressing patient values in the design of cardiac technologies. Similarly, Ramachandran et al [54] emphasize in their review that considering the needs and preferences of patients during the initial stages of program design can help alleviate issues with poor implementation and adoption of CTR. Although the values of patients with cardiac conditions as a foundation for eHealth design have been identified in recent work by Bente et al [50], these values are generic (eg, “To have or maintain a healthy lifestyle”); do not explore specific behaviors tackled by CR, such as exercise behavior or coping mechanisms; and do not address the athlete subpopulations, whose objectives might differ from those of the general population. In the following sections, we present our investigation into the value-centric design of CTR technologies for athletes.

## Methods

### Study Rationale and Design Overview

We conducted a qualitative study guided by the HCD paradigm and the participatory health research (PHR) paradigm. The HCD paradigm implies that design activity should prioritize defining the meaning that a product, system, or service offers to people, focusing on *motivation, discourse, and learning before addressing implementation* [82]. It relies on techniques that *engage, interact with, and empathize with people*, uncovering needs, desires, and experiences beyond their own awareness [82]. Similarly, but more focused on the knowledge creation process within a health care problem, the PHR paradigm is a collaborative research approach that actively engages stakeholders as cocreators of knowledge throughout the research process, ensuring greater relevance, applicability, and impact of the findings [83].

To operationalize this participatory approach, the VSD framework was used to identify and embed stakeholders’ values into the design of CTR interventions. VSD recognizes the interplay between human experiences and technology, where values are both embedded in and influenced by technology [36]. CTR, deeply reliant on technologies, impacts users’ daily lives, from private digital consultations to continuous heart rate (HR) monitoring during routine activities [4]. Thus, CTR designers must understand and incorporate what users value in their everyday lives to create interventions that seamlessly align with and support those values. Recent research by Cruz-Martínez et al [32] and Tadas and Coyle [48] highlights the need for incorporating patients’ values into designing cardiac technologies to enhance sustainable self-management, technology adherence, and intervention effectiveness.

We used participatory qualitative methods that align with tools used in HCD and PHR (eg, card sorting and FGs) and are rooted in the VSD framework (eg, value-oriented interviews and stakeholder identification sessions). We elaborate on these methods in the following subsections.

**Figure 1:**
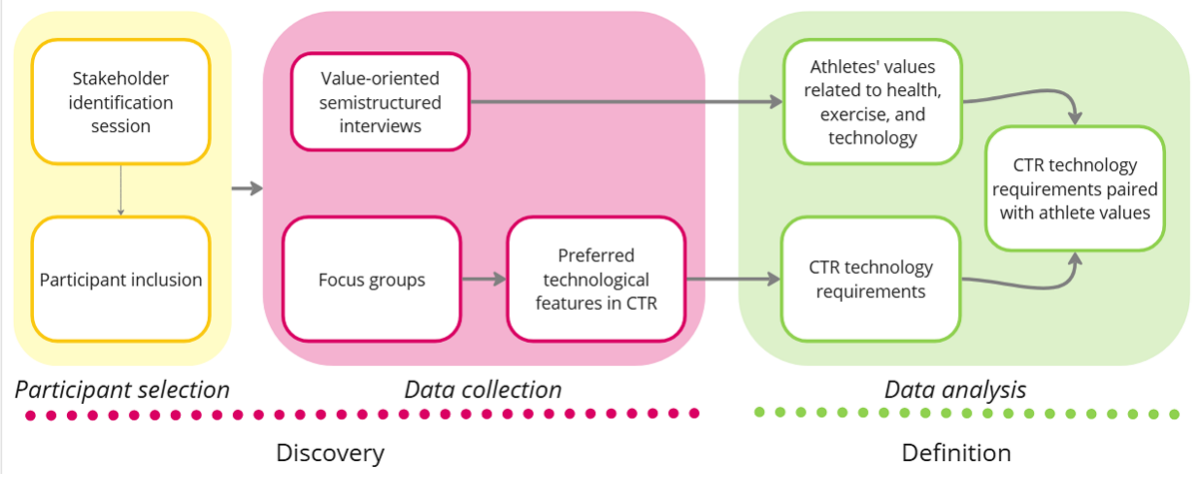
The structure of our study divided into two phases: (1) discovery, including participant selection and data collection; and (2) definition, including data analysis. CTR: cardiac telerehabilitation.

To achieve our research goals (ie, elicit athletes’ values in CR, including health and behavior tracking, and define design requirements for CTR technologies addressing athlete values), our study comprised two main phases: (1) *a discovery phase*, including key stakeholder identification and recruitment and data collection through 25 value-oriented semistructured interviews and 6 card-sorting FGs; and (2) *a definition phase* in which, through data analysis, the values (elicited in the interviews) were defined and matched with the identified CTR requirements (derived from the FG results; Figure 1).

### Ethical Considerations

This work was conducted by 2 Dutch institutions: Eindhoven University of Technology in collaboration with Máxima Medical Center Veldhoven—a teaching hospital that offers both conventional in-hospital CR and CTR. The study protocol was reviewed by both the university’s ethical committee and Máxima Medical Center’s medical ethical committee and received a waiver that ethics approval was not required. The collected data were stored and processed according to the data policies and agreements of Eindhoven University of Technology. Informed consent was obtained from all participants, and each participant received a €20 (US $22.07) gift card as compensation.

### Stakeholder Identification Session and Participant Selection and Recruitment

VSD posits that technologies—in this case, CTR technologies—have real and sometimes nonobvious impacts on those who are directly or indirectly affected by them [85]. VSD encourages designers to identify a robust set of direct and indirect stakeholders at the beginning of the design process and legitimate those likely most affected and who should be included in the design process [78]. To identify stakeholders in addition to athletes in the context of CR, we first conducted a stakeholder identification session using the IGOHcaps method [86]. This method seeks to identify health care actors (or stakeholders) in a dynamic environment. As CR is a complex, multifaceted system of health care services that changes its dynamic over a long period (ie, from hospitalization to self-management and further), the IGOHcaps method was considered suitable for identifying the right participants for our study. The stakeholder identification session lasted 60 minutes and involved an eHealth designer and 5 clinicians working in CR: 1 (20%) cardiac nurse practitioner, 2 (40%) cardiologists, 1 (20%) sports physician, and 1 (20%) resident sports physician.

The IGOHcaps method proposes identifying health care actors as *humans* or *organizations* that *provide*, *control*, *support*, or *accept* health care services (ie, rehabilitation or telerehabilitation). A set of well-defined guidelines are used in the process, such as “actors depend on a specific context and time frame” [86]. On the basis of this method, participants were first asked to identify as many actors as possible using Post-its on big posters in 10-minute sessions per category (ie, providers, controllers, supporters, and acceptors). Finally, participants put all the identified actors together, removed redundancies, and added missing actors, creating a broad overview of direct and indirect stakeholders involved in CR and CTR.

On the basis of the outcomes of the stakeholder identification session, we selected actors fitting the following criteria: (1) *human providers*—directly impacted by CTR technologies and in direct contact with athletes—related to cardiac health (eg, cardiologists), exercising (eg, physiotherapists), psychosocial therapies (eg, psychologists), and general rehabilitation (eg, nurse practitioners; Table 1 and Textbox 1) and (2) *athletes with established CAD* (human acceptors) who exercise >4 hours per week and finished CR within at most 1 year before the moment of the interview (Table 2). In the end, 15 athletes and 12 HCPs were included. The number of participants aligns with recent research suggesting that 9 to 17 [87] or 15 to 30 [88] participants can be sufficient to reach data saturation in qualitative studies that deal with homogeneous populations (in our case, athletes with cardiac conditions and HCPs working in CR), narrowly defined objectives (in our case, CTR requirements for athletes with cardiac conditions) [87], and a structured interview guide [88].

**Table 1:** Other stakeholders (health care professionals; HCPs) participating in our study^a^.

HCP ID	Experience (y)	Expertise
HCP1	27	Sports physician
HCP2	3	Physiotherapist
HCP3	6	Sports physician in training
HCP4	16	Clinical psychologist
HCP5	14	Nurse practitioner in CR^b^
HCP6	13	Exercise instructor (department of physiotherapy) and lifestyle coach
HCP7	27	Primary care physiotherapist
HCP8	>20	Physiotherapist
HCP9	12	Cardiologist
HCP10	10	Nurse practitioner in CR
HCP11	19	Cardiologist
HCP12	8	Nurse practitioner in CR

^a^HCPs 1, 2, 3, 7, and 8 participated in both the focus groups and interviews, HCPs 11 and 12 only participated in the focus groups, and the rest of the HCPs participated only in the interviews.

^b^CR: cardiac rehabilitation.

**The roles of participating health care professionals in the rehabilitation trajectory.**

- Sports physician: conduct of cardiopulmonary exercise testing, formulation of training recommendations (including any restrictions), consultations for discussion of test results and recommendations, and supervision of results of exercise stress tests [89]
- Physiotherapist: supervision of the individually tailored exercise training program and support for a physically active lifestyle
- Clinical psychologist: assessment of the psychiatric problems (that were already present or developed after the cardiac event), supervision of psychoeducational prevention modules (group sessions), and individual treatment of patients if necessary
- Nurse practitioner in cardiac rehabilitation (CR): case manager (ie, patient advocate that supports, guides, and coordinates patient care), intake session for discussion of lifestyle behavior and changes and individual goals, performance of assessments for risk of anxiety and depression, and end evaluation of CR program
- Exercise instructor (department of physiotherapy): supervision of the individually tailored exercise training program and support for a physically active lifestyle (similarly to a physiotherapist)
- Cardiologist: treating physician of patients with cardiac conditions, referral for CR, medication optimization, and risk assessment

**Table 2:** Athletes (patients) who participated in our study; all athletes participated in both the interviews and focus groups except for P1 (absence due to injury) and P9 (lack of time availability), who only took part in the interviews.

Patient ID	Gender	Age (y)	Employment status; family and living situation	Sports practiced	Self-monitoring technology used	Time since finishing the program
P1	Male	76	Retired; living with spouse	Tennis, cycling, and fitness	Chest strap HR^a^ monitor	Just finished the program
P2	Male	70	Retired; living with spouse	Table tennis and cycling	Bicycle computer, chest strap HR monitor, and blood pressure monitor	5 months
P3	Male	44	Employed; living with spouse	Mountain biking, running, boxing, and fitness	Smartwatch and ChatGPT (for training schemes)	Just finished the program
P4	Male	65	Retired; living with spouse	Cycling	Smartwatch, chest strap HR monitor, bicycle computer, Strava app, and blood pressure monitor	4 months
P5	Male	75	Retired; living with spouse	Tennis, padel, and golf	Golf watch	1 month
P6	Male	79	Retired; living alone and close to his children	Running and competitions (eg, track races)	Smartphone step counter	1 year
P7	Male	46	Employed; living alone and close to his parents	Strength training and cycling	Fitness watch and cycling mobile app	Just finished the program
P8	Male	57	Employed; living with spouse and children	Running (competitive), tennis, and mountain biking	Fitness watch, mobile app, and bicycle computer	10 months
P9	Male	54	Employed; living with spouse and children	Running and high-intensity training	Fitness watch and mobile app	7 months
P10	Male	57	Employed; living with spouse and children	Mountain biking and spinning	Fitness watch, mobile app, and chest strap HR monitor	2 months
P11	Male	58	Employed; living alone and close to his siblings	Fitness, cardiovascular fitness, and high-intensity training	Blood pressure monitor and smartwatch	2 months (did not participate in the physical training program)
P12	Male	65	Employed; living with spouse	Running, mountain biking, and padel	Fitness watch	4 months
P13	Female	73	Retired; living with spouse	Cardiovascular fitness, strength training, and spinning	Bicycle computer	Just finished the program
P14	Male	62	Employed; living with spouse	Fitness training, bodybuilding, and cycling	Smartwatch	6 months (did not participate in the physical training program)
P15	Male	68	Retired; living with spouse	Running, competitions (marathons and ultramarathons), and cycling	Fitness watch and chest strap HR monitor	9 months

^a^HR: heart rate.

Patient recruitment was conducted over several weeks via the hospital by a clinician (second researcher), whereas HCPs were recruited through the extended networks of the project’s members from the same hospital.

### Semistructured Interviews

To incorporate athletes’ values into technologies, we first needed to identify these values. To do so, we conducted 25 individual, semistructured interviews with 15 athletes (P1-P15; Table 2) and 10 HCPs (HCP1-HCP10; Table 1). Each interview was conducted following 2 variations of an interview script (one for athletes and one for HCPs) developed by looking at recent value-oriented studies [50,81]. The interview guides explored themes such as the value of physical activity in athletes’ lives, good and bad experiences with CR, information flows and relationships with HCPs, social support, and technology used for managing sports and health (Multimedia Appendix 1).

In total, 2 researchers conducted the interviews, which lasted 60 to 70 minutes and were audio recorded. Recordings were used for verbatim transcriptions and translations from Dutch into English.

### Card-Sorting FGs

One significant assumption of the VSD framework is that certain technologies or tools support certain values more promptly while being less suitable to address others [77]. This suggests that more or less suitable “properties” or “features” can be designed into technologies to address specific human values. As mentioned in the Related Work section, CTR interventions are complex systems comprising health care services (such as education, coaching, and consultations) delivered through multimodal technologies—often a combination of (commercial) wearable sensors and digital (mobile or desktop) applications [5,90]. We aimed to derive high-level design recommendations for CTR technologies; however, we included both technology features and care service features as they heavily depend on each other—care service features dictate the content and features of the technologies used (eg, designing a platform monitoring individual sports training is different from designing a platform monitoring synchronous group training). Nonetheless, we stressed the importance of technology to our participants during the card-sorting activity—while some cards pertained to care service features (eg, “to have face-to-face consultations”), participants were asked about ways in which technology could mediate or enable those features.

To further explore desired CTR features that could potentially support athletes in rehabilitation, 6 FGs were held. A total of 87% (13/15) of the athletes who participated in the interviews took part in the FGs. Athletes were divided equally into 4 FGs (FG 1 had 4 participants). HCPs 1, 2, 3, 7, 8, 11, and 12 were divided into 2 additional FGs (FG 5 with 3/7, 43% of the participants and FG 6 with 4/7, 57% of the participants). Each FG lasted 60 to 100 minutes and was facilitated by a clinician and a designer. The FGs were exploratory and creative and applied participatory methods from design thinking [91]. All 6 FGs were given the same assignment—a closed card-sorting task of features of CTR interventions. Card sorting is a method that helps understand the people we are designing for [92]. In our case, we aimed to understand what athletes would find most important and useful in CTR that suited their values. Therefore, HCPs were asked to respond from the athletes’ perspective.

We extracted features from recent studies reviewing telerehabilitation technologies [4,5,8,18,61] as well as reviews of cardiac self-management eHealth and telemonitoring [32,37]. To reduce the complexity of the resulting list of extracted features, they were divided into 4 main categories based on the composition of telerehabilitation systems, as described in the Related Work section: *education and assistance*; *consultations, coaching, and guidance*; *monitoring behaviors for supervision and oneself*; and *coexperience (social aspect)*. Because the existing categories were still broad and lacked visual structure, each category was divided into subcategories based on the user journey proposal by Knoche et al [84] of patients using intelligent data-driven health technologies (Multimedia Appendix 2). In addition, we provided empty cards for each category for extra suggestions.

**Figure 2:**
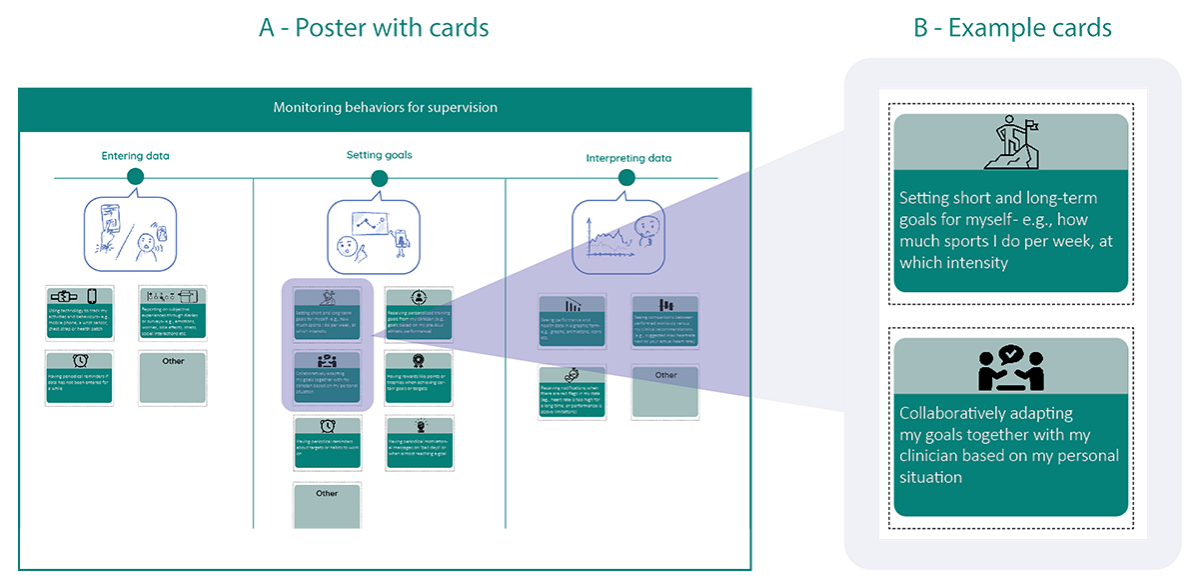
(A) Poster with cards related to monitoring behaviors; (B) 2 example cards zoomed in. Sketches depicting subcategories (eg, setting goals) were inspired by the user journey of a patient interacting with intelligent health interfaces [92].

**Figure 3:**
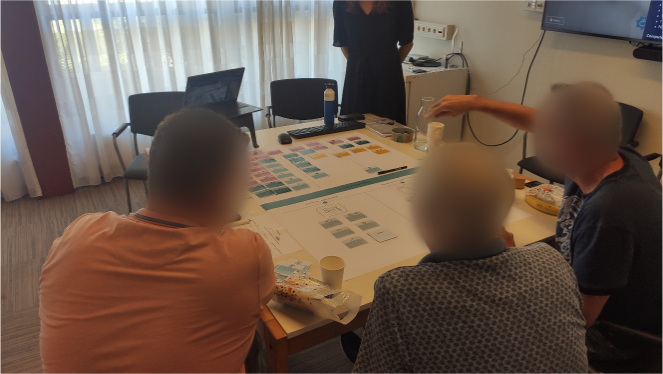
An example depicting card-sorting activity outcomes from focus group 1—cards from different categories sorted as "must have," "nice to have," and "not needed" from left to right.

**Figure 4:**
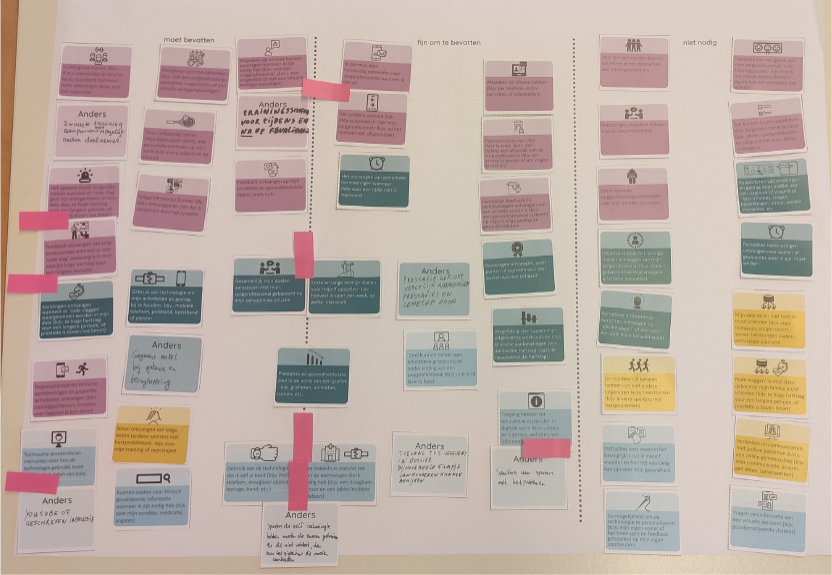
Focus group 4—patients interacting with the cards during the card-sorting session.

The 4 categories were presented on posters containing individual cards depicting technology features (Figure 2 [84]). Participants were asked to work together to sort cards one by one on a canvas divided into 3 sections (ie, *must have*, *nice to have*, and *not needed*; Figures 3 and 4). Participants were asked to explain the motivation for their choice for each card, encouraging group discussions. The entire interaction was audio and video recorded with the consent of the participants.

### Data Analysis: Defining Values and Matching Design Requirements

#### Overview

To ensure transparency and minimize researcher bias, the data analysis process was iterative and collaborative. Both the interview and FG recordings were initially auto-transcribed using Amberscript (Amberscript Global B.V.). The transcriptions were then reviewed alongside the recordings to correct errors from the automatic transcription process and subsequently translated. Thematic analysis was conducted following the 6-step framework by Braun and Clarke [93] to identify and analyze patterns in the data. First, the researchers familiarized themselves with the data by reading the transcripts and notes taken during the interviews and FGs multiple times.

#### Defining Athletes’ Values

IBS analyzed the interview transcriptions (n=25) to identify quotations about what athletes with CAD find important in life and care. Inductive coding was used to identify codes related to athletes’ individual needs regarding exercising, CR, and lifestyle. These codes were grouped under subthemes illustrating more generic needs of the group (eg, “exercise support for one’s fitness level”). LF checked the identified needs for analysis validation. To define values, we followed their VSD definition of being *more generic ideals or drivers of athletes’ needs* [50,79]. Consequently, according to the VSD methods, we developed a value-oriented coding manual by assigning a small description to each initial code related to a need (by rereading the data; Multimedia Appendix 3). Afterward, the needs were grouped based on underlying themes (ie, desires) that could drive the specific needs—the values. LF verified the links between needs and values, and the coding manual together with representative codes were discussed among the group of authors. Codes that did not directly refer to athletes’ needs (eg, “miscommunication between healthcare professionals”) were grouped under non–value-related themes and, because of the scope of this work, were not emphasized in this paper.

#### Sorting Preferred CTR Features

The results of the card-sorting activity were organized based on the preferences of each FG—each feature received a score (2—*must have*, 1—*nice to have*, and 0—*not needed*) and was ordered from highest scoring to lowest scoring. If participants could not agree on a category, the scores were averaged. By ordering them by score in Microsoft Excel, we identified preferred CTR features from the most desired (highest scoring) to the least desired (lowest scoring). Participants’ observations during the FGs (either recorded or written on cards) were noted next to each corresponding feature to be used in the requirement definition phase.

#### Defining CTR Requirements Paired With Values

The features (cards) that were a “must have” for at least 2 FGs together with participant observations were clustered under themes representing requirements. Occasionally, participants within a group held differing opinions on specific features—some felt that they did not need a feature, whereas others felt that it must be included. We opted to incorporate disputed features as requirements if they provided substantial value to certain participants. For example, one athlete whose family resided at a distance and lacked a strong local social support system considered sharing data with family crucial. In contrast, other participants who lived with family did not consider this feature desirable. Given the importance of this feature for the peace of mind and safety of some athletes, we included data sharing with family as a requirement.

Through small iterations during the analysis process, the emerging requirements were linked to one or more identified values. This enabled an iterative reflective process of requirement adaptation according to the expressed needs and values of athletes. These phases were conducted by IBȘ, with LF consistently checking after each iteration. The resulting list of requirements connected with values was checked and discussed among the rest of the research team. This led to defining high-level requirements and guidelines for a technology-mediated CTR intervention that could support athlete values. Preferences and requirements about improvements to colocated CR services also surfaced in our FGs. As these insights are out of the scope of our study, they will be mentioned but not discussed in this paper.

### Reflexivity and Research Positionality Statement

To explore how CTR technologies can align with the values and needs of athletic patients, this study represented a collaborative effort between HCI researchers specializing in the social implications of interactive systems and human-data interaction in health and broader contexts and clinicians with expertise in sports cardiology and CR. While this work is focused on technology design, the second author (ie, clinician in training) was actively involved in all stages of the research, ensuring the clinical validity of the derived conclusions as well as the ethical integrity of the research.

Reflexivity was integral to mitigating biases and enhancing credibility throughout the research process. We recognized the potential influence of our previous knowledge and assumptions on study design and data interpretation. To address this, we conducted collaborative discussions with team members not involved in data collection or initial analysis to challenge assumptions and refine the design. The stakeholder identification meeting of clinicians outside the research team ensured diverse views in participant selection. Furthermore, data collection was conducted collaboratively with multiple researchers present, enabling diverse observations, reducing individual bias, and fostering critical reflection through team debriefs. The iterative data analysis enabled cross-checking of the themes and values by multiple researchers to validate findings and ensure coherence. Finally, the triangulation of perspectives achieved by including both athletic patients and diverse HCPs minimized bias by incorporating varied experiences. This approach ensured a balanced and rigorous examination of the data.

## Results

This section contains identified athlete values derived from the interviews, card-sorting outcomes, and CTR requirements derived from the card-sorting activity paired with athletes’ values.

### Semistructured Interviews: Values of Athletes Regarding CR

#### Overview

The results from the interviews conducted with athletes and other stakeholders (n=25) revealed the presence of 12 key themes encapsulating athletes’ values within the context of CR and the broader context of personal health management. Each of these key values (ie, high-level desire) was generally supported by multiple identified needs (ie, demands described by participants concerning CR participation and health management). In some instances, a single need could be associated with multiple values. For instance, the need to “observe progress in one’s physical performance during the recovery process” could be interconnected with values such as “a goal and performance-oriented approach” and “health and performance quantification.” Moreover, the identified values represented the overarching desires of athletes, whereas some needs might only pertain to some individuals. The subsequent sections will provide insights into findings related to each of the 12 identified key values clustered in 3 groups: *body centric*, *care centric*, and *data and technology centric*. An overview of the values and needs and example quotes can be found in Multimedia Appendix 4.

#### Body-Centric Values

##### A Dynamic Lifestyle

Athletes were individuals who placed a high value on physical activity and exercise. Participants viewed exercise as part of their identity, a way to cope with psychological struggles, a way to compete with oneself and push one’s limit, and a way of being part of a community when it comes to social sports. However, of utmost significance, all participants valued maintaining a healthy lifestyle and linked exercise and sports with preserving vitality and good health. All participants positioned exercising highly among life values such as being with family and enjoying work and life. The lack of CR programs addressing the sports they valued hindered their enjoyment and motivation to participate:

That’s really important—if I can do sports, that’s the foundation of the day. Then I can move forward...Maybe [I like] the adrenaline, I've always been active. Working on the farm from primary school, always at work, worked at the bakery, always training. I was at the athletics club in [city name], I went there twice a week. I have always been active...[After the cardiac event] I had the fear that I could no longer exercise. That's a pretty awful though. If I can't exercise anymore, what am I supposed to do?

P6

##### Independence and Confidence in One’s Body

Athletes valued their individuality, independence, and self-efficacy. They were very sure of themselves and did not like to doubt their capacity to recover and return to their athletic capabilities and previous lifestyle. They expressed confidence in their recovery journey and relied less on clinicians, valuing their opinions alongside their own ability to discern their body’s needs. Athletes knew the value of physical activity and took pride in remaining fit despite older age. Consequently, they did not need external motivation. Still, they needed facilitators to understand their limits and apply the clinical knowledge in the home environment, where they wished to safely exercise similarly to before hospitalization. Although very confident in “feeling” what their body needed, the CAD diagnosis could unbalance their “internal compass”:

I’ve always had a positive perspective on life...I immediately thought “This [ie, heart attack] just happens to you and we’re going to make the best of it. Seriously work on it.” I have tried very hard to function properly again. And I don’t suffer anymore...I feel myself if I really go too far...She [ie, the cardiologist] said “You can just exercise, of course, you should never go crazy. That’s never good. But no restrictions.”...She gave me that confidence.

P14

##### Coming to Terms With One’s Condition

Most participants described the diagnosis as unexpected due to their healthy lifestyle and absence of symptoms. The lack of control over their diagnosis, often with a family history of cardiac conditions, coupled with sudden fatigue and loss of physical fitness, had the potential to profoundly affect their and their family’s outlook on life. Athletes had high ambitions of returning to their previous exercise capacity and found difficulty in accepting a “failing body.” At the onset of CR, it was crucial for athletes to cultivate coping strategies to interpret their body’s signals. Clear exercise limitations, such as maximum HR, helped athletes establish boundaries. They required guidance on managing extreme fatigue and knowing when to pause and rest. In addition, athletes prioritized resolving frictions with their families regarding safe exercise and activity levels. They achieved this by negotiating the specifics of where, when, and how much exercise they engaged in. Their ultimate objective was to return to the routine and normality of everyday life:

You actually want to pedal higher wattages, as much as possible...I look next to me, to a colleague who had been practicing for a while. It was on 106. I thought “I should be able to do that too,” but that was quite disappointing...You’re on a racing bike. You are very fast on such a thing, and very far away...I always have my phone and things with me, data, so they [ie, family] can find me...I have that app from my odometer—it also tells my son where I am at that moment...those are the things I take with me to be able to cycle safely.

P4

#### Care-Centric Values

##### A Goal- and Performance-Oriented Approach

Athletes found goals and performance important during the CR program. One crucial need was to return to previous athletic capabilities by having clear, progressive performance targets. Health and athletic goals intertwined, for instance, reaching target HRs and regaining the ability to mountain cycle. They valued pushing their limits and making steady progress alongside clinical validation of their advancements. As a result, some participants found the exercise program too easy and the CR intervention less effective. They suggested adjusting exercises to match their intensity. Other patients found usefulness in regaining confidence under professional supervision. To reach their goals, some athletes used resources within their network, such as personal trainers, friends, or relatives who were HCPs:

It [ie, the exercise program] didn’t make much sense...It turned out that my condition was more than sufficient. Then the real question is “why are we doing this?”...They actually slowed you down rather than stimulating you...It didn’t really help much. I honestly found it slowing me down a lot. Because I have to cycle for fifteen minutes to get here [ie, hospital] and fifteen minutes back. And then I actually cycle even more intensively [commuting] than there [ie, at the exercise program].

P2

##### Concise, Actionable Guidelines

A significant number of athletes felt like they had been “left in a void” during CR—clinicians did not pay enough attention to their needs because athletes already knew the value of exercise. Most athletes expressed the need for clear, preferably quantifiable indications of exercise limitations (eg, the maximum HR they could reach), personalized variations of sport-specific training schemes that made them feel safe but performant, and education on the influence of medication on performance. Some athletes found open-ended exercise limitations (eg, “Do whatever feels good”) enough for safe sport resumption, whereas others found them confusing and insufficient:

I still find it difficult to understand, what is very good with the beta blockers [ie, medication used for secondary prevention].... It’s still a difficult process to find out where to be in your exercise. And if they could have given some guidance in that, what can you do maybe with intervals, like boxing, which is very high interval training and where should you be. Should you be careful with some things or with your HR, or yeah, they didn’t think much about it.

P3

##### Trustworthy, Readily Available Support

Athletes expressed their challenges and objectives adeptly, valuing clinicians who offered clear, concise, and actionable solutions. They sought easily accessible health care services for emergencies or alarming symptoms—some had needed rescue during mountain cycling or gym workouts. They appreciated impromptu talks with physiotherapists during training and desired swift access to clinicians for direct, sincere responses to inquiries without delays encountered in current e-consultation systems. Moreover, athletes appreciated clinicians who gave patients control over health-related decisions and involved them in the decision-making process regarding their health journey:

If I send it [ie, exercise recommendations] via email, it will quickly reach the person. We’ve had a conversation [ie, consultation] and I’ll fill that in a bit afterward. It is annoying for people if they have to wait two weeks for a letter containing those recommendations. They have just received that advice, and then they actually can read it that evening, so I will send it that way.

HCP3

##### Care With a “Personal Touch”

Although athletes were very open to exercising independently in the home environment and communicating from a distance with HCPs, they still appreciated personal approaches to health care. Many athletes mentioned that they did not want to feel or be treated like a number and wanted to maintain face-to-face contact and human interrelations periodically during the rehabilitation period. Moreover, for some, there was a significant dip in morale after hospitalization, and empathy and reassurance were highly needed. Some patients described a lack of empathy from HCPs for being “fitter” than the general population and having better chances to recover:

When I had a conversation with the nurse practitioner I noticed she was not serious. She said “Within 3 weeks you will forget you had a heart attack.” It was a pity for me, so I was sad about it. I thought to myself—what do I have to do now? Where are the people? How can I find the people? But I was afraid to call the nurse to ask where because they were so busy. Then I think it’s a stupid question.

P11

##### In- and Outside-Hospital Oversight

Athletes were insecure about new exercise limitations, how much they could push themselves, and exercising safely outside the hospital environment. They mentioned being supervised as crucial for their confidence and reassurance, especially in the first few months after hospitalization. Within current CR, athletes participated in exercise programs not for lifestyle changes per se but to feel supervised by HCPs and have the feeling that they were doing the best they could for their health. Being supervised provided reassurance, security, confidence, and a feeling of being cared for:

I think in the beginning, it’s quite scary to do heavy exercise without any supervision. The defibrillator should do its job when there are hard issues. I think after the hospitalization you need some reassurance from somebody looking after you if there any issues.

P3

##### Emotional Support and Sharing

Athletes did not need social support to maintain healthy behaviors. However, they appreciated the emotional support and opportunity to share their struggles and anxieties with their family and close ones but also with HCPs such as the clinical psychologist. While not essential, they valued the bonding opportunity of conversing with other patients with cardiac conditions. However, many participants did not encounter fellow athletes in their CR group. Participating in the FG provided a pleasant chance for them to share treatment options, experiences, and their common passion for exercise:

I think besides the rehabilitation program, the physical part, it was also nice to speak with people who also had an [heart] attack. Just to hear some experiences or like the medication I used—I had a lot of side effects and they said “Well, I also had that. But after a while it went away, or I tried another one or asked for injections.” This was nice.

P10

#### Data- and Technology-Centric Values

##### Health and Performance Quantification

Athletes had advanced data literacy, often using wearable sensors, bicycle computers, or mobile phones to track metrics such as HR, distance, speed, and time. However, a few participants preferred relying on their body awareness over sensing technology. In their rehabilitation, athletes diligently conducted routine checks such as blood pressure and HR measurements along with hospital tests. One participant even maintained a Microsoft Excel sheet in which they recorded their blood pressure daily. After diagnosis, some athletes’ relationship with wearable sensors shifted—they used data to reassure themselves about their health and recovery. They also valued progress reports to gauge recovery, correlating their body’s feedback with measurable improvements such as lifting more weight or running faster:

Interviewer: What do you like about your fitness watch?

P9: The monitoring it does, so you know anything like HR, but also the exercises themselves and seeing progress—that improves and then you are really happy. That's something I saw, there was a tremendous drop [ie, in fitness] when I had surgery and the months before. It dropped significantly and now you see it. And now it's higher than even before the surgery. So that's really motivating, especially those things that you have to really work hard for to, you know, get one level up.

##### Clinical Validation of Information and Data

Some athletes valued data for performance measurements, but trust in wearable data diminished after diagnosis. One participant’s distrust stemmed from the wearable’s failure to detect their myocardial infarction. Consequently, athletes placed importance on clinicians giving meaning and validating data on vital signs. Athletes considered it crucial to discuss various measurements such blood pressure, HR (eg, variability and zones), and performance metrics with their clinicians. They sought to derive meaning from these measurements in the context of their cardiac condition or medication intake. Some athletes remembered sharing data overviews from fitness apps with their clinicians during consultations but acknowledged receiving little substantive feedback due to the brevity of the consultations. Moreover, athletes found it significant that the information they received about their recovery process, cardiac intervention, or medication came from clinicians, not from other sources such as the internet or other people:

The most important thing for me was measuring [ie, during CR]. Things are tested—what you do, and then you end up with that bike test again. Measuring is knowing, I say, that was very important to me in that rehabilitation...I have to do it and measure it, and the healthcare provider can judge. What does that mean now, is that good or should I do something else? That is difficult by yourself...I sometimes go to the doctor and then he sometimes says “take a look at ‘Thuisarts’” [ie, a website with general healthcare knowledge]. But I’m not into it. If there is something wrong with me, I want an expert who can tell me something.

P5

##### Reliable Information Systems

Even though athletes were willing to be supervised from a distance, exercise in their home environment, and communicate with clinicians through digital channels, they did require reliable information systems that facilitated the process instead of burdening it with technical issues. One important consideration was reliable sensors and data streams—some athletes did not fully trust data from commercial wearables and used additional devices (eg, chest strap and blood pressure monitor). Moreover, they underlined the importance of reliable infrastructures that can transmit data and information to and from clinicians without delay. They also stressed the importance of user-friendly workout schedules that could be easily implemented with technologies they already used:

[The most important thing to consider when developing a CTR system] To know that you have a good connection. The rest will take care of itself...The moment I step on that [ie, exercise bike], that data also ends up in the hospital...it could end up on a server in the hospital. You can, so to speak, watch it a few days later and then give feedback.

P1

### Card-Sorting FGs: Preferred Features of CTR Systems

#### Overview

The card-sorting activity allowed participants to present an overview of desirable or undesirable CTR features for athletes. The characterization among *must have*, *nice to have*, and *not needed* differed from group to group based on their personal needs, group dynamic, composition (patients vs HCPs), perceived independence in one’s health management journey, and experience with technology. We present a summary of the card-sorting results in the following sections and in full in Multimedia Appendix 5.

#### Consultations, Coaching, and Guidance

Participants considered periodic checks (either through electronic consultations or surveys), remote supervision of sport-specific training, and personalized content comprising sport-specific actionable guidelines as *must haves*. The detection of alarming symptoms in the collected data was deemed very important, as was receiving actionable feedback on how to react to these symptoms or prevent them, especially during intense exercise. Frictions arose among athletes regarding remotely supervised group training—most participants considered it essential at the beginning of the program, whereas 15% (2/13) of the athletes believed that independent training was best as they could “go at their own pace.” In-person consultations were a *must have* except in FG 1, whose participants deemed them unnecessary as long as remote consultations were provided. A system that facilitated communication about exercise data between the athlete and HCPs and athlete-HCP feedback on training recommendations was considered a *must have* or *nice to have*. Athletes had varying opinions on monitoring emotions and receiving emotional support from clinicians. Some viewed it as essential, especially when struggling to accept their condition, whereas others believed that emotional support was better provided by family or friends. Finally, recommendations from virtual assistants (eg, automated recommendation systems) were generally ill-favored because of a lack of trust in their reliability.

#### Monitoring Behaviors for Supervision and Oneself

Participants felt confident about receiving notifications when irregularities in their data were detected. Most participants preferred wearable sensor monitoring of physical activity to subjective self-reporting mostly because of the trust in and objectivity of sensor data, as well as the familiarity with and pragmatic benefits of automatic data collection. Nonetheless, some participants recognized the added value of subjective data collection for clinical decision-making and depersonalization of data and for expressing anxieties and insecurities related to data inaccuracies and safe sports. Athletes underlined the importance of support for collaboratively adapting individually set performance goals with HCPs. Receiving personalized goals from clinicians was disagreed upon—some athletes felt confident in setting their own goals, whereas others felt that clinical guidance was essential at the beginning of rehabilitation until confidence in one’s athletic abilities was regained. Graphical representations of performance and health data, as well as parallels between actual performance and clinical recommendations, were labeled as *must have* or *nice to have*. In line with case studies describing experiences of athletes with CR [2,75,91], our participants expressed little to no interest in motivational queues, rewards, and reminders because they already felt motivated enough to rehabilitate (exercising but also eating healthily and taking medication).

#### Education and Assistance

Technical assistance and instructions on using CTR technologies, as well as having the possibility of integrating their own wearable sensors into the monitoring system for familiarity, convenience, and trust, were deemed a *must have* by all participants—HCPs underlined that, at times, athletes used sensors that were superior to the equipment provided by the hospital. Access to digital information about being an athlete with CAD was important to most participants as similar reliable resources were not available online. Receiving information from virtual assistants (eg, chatbots) was not needed because of a lack of trust in the clinical validity of the information—some clinicians regarded it as a nice addition that could alleviate the workload of HCPs. Similarly, some participants considered information about why monitoring is beneficial for health as common knowledge and, therefore, not necessary.

#### Coexperience (Social Aspect)

Peer-based support was appreciated only within the context of the program and, preferably, in person. Online communication with peers was not viewed as important as athletes felt confident in themselves and their existing support systems and expressed that they would not make use of online communities. Sharing data with family also obtained a low score, with the exception of 15% (2/13) of the participants, who already shared their location with loved ones for safety, alleviating anxiety, and shared decision-making. Virtual environments that facilitate training with peers were not highly valued as athletes already had social groups to train with or preferred training individually.

### High-Level CTR Technology Requirements Paired With Athlete Values

The derived requirements indicate that technology can facilitate exercise monitoring; the interaction among patients, clinicians, and the monitored data; and mechanisms of remote coaching and remote consultations. The independence that athletes exhibited in their health care journey was echoed in the resulting specifications, which underscored their inclination to personalize and monitor their physical and recovery progress independently, diverging from standardized exercise regimes. The pragmatism and data-centric approach were evident in the need for precise exercise restrictions, recommendations, and progress measurements. Athletes prioritized a straightforward and informative system over motivational strategies such as gamification or reward systems. The dynamic and disciplined lifestyle that they associated with their well-being and identity manifested in most of the technology requirements. However, glimpses of the desired personal touch emerged in open data-gathering approaches or the request for individualized sport-specific feedback. The derived CTR technology requirements can be found in Textbox 2, and an extended version containing paired athlete values can be found in Multimedia Appendix 6.

Moreover, although participants were encouraged to brainstorm ways in which technology could augment health care services, *technology was not consistently the preferred choice*. These insights are valuable for the HCI community as they first identify the preference for a hybrid CTR intervention, and second, they elucidate user scenarios in which technology should not be imposed as a solution, thereby averting user frustrations or resistance. Participants found face-to-face mediation in colocated contexts most effective for the group, emotional support, or trust-building interactions. For instance, while peer support was appreciated in person, it was not preferred in online groups. Similarly, at the beginning of the program, in-person clinical consultations, group education sessions, and standardized exercise sessions were preferred for building confidence and bonding. Finally, open communication was preferred over technology for social support in the home environment. These requirements relate to the values of *care with a “personal touch”* and *emotional support and sharing* as participants emphasized the importance of occasional human interaction in feeling supported by a health care system that prioritizes compassion and human connection. We used these insights to advance knowledge about technology-centric requirements in the context of CTR for athletes.

**Derived cardiac telerehabilitation technology requirements.**

**Supporting remote monitoring**

- Remotely monitored sport-specific training (eg, type of sport, duration, frequency, heart rate [HR; zones], and degree of effort) and biophysical measurements (eg, blood pressure)—before and during the cardiac rehabilitation program
- Exercise data collection and aggregation from athletes’ own wearable sensors (eg, sports watches, chest straps, and mobile apps); the hospital provides patients with sensors in case they do not use one
- Simple, brief, open approach to subjective data collection gathering contextual information (eg, symptom uncertainty, sport-related anxiety, and questions about sensor data irregularities)
- Technical instructions and online assistance in monitoring, syncing, sharing, and correctly interpreting the data (eg, instructional videos or written information)
- Continuous and automatic transmission of remote data to clinicians; opting for manual, discrete transmission is possible

**Supporting human-data interaction**

- Graphs displaying training progress (eg, HR and intensity minutes) and sessions (eg, duration and HR zones) contrasting with limitations and recommendations (eg, exceeding maximum HR) for easy understanding by trainers, family, and clinicians
- Instant alerts for exceeding clinical limitations during training (eg, excessive time spent in HR zone 4)
- Alerts for prolonged anomalies in the data (eg, exceeding limitations and signs of cardiac arrhythmia) with follow-up recommendations for action—either adjusting performance or seeking clinical assistance
- Selecting and sharing monitored data snippets (eg, HR from a specific exercise session) with clinicians within the system, facilitating remote data-focused discussions
- An overview of clinical test results such as exercise stress tests and clinical scans
- Sharing health and exercise data with family, including exercise location and alarming signals, for discussion, negotiation, and easing concerns

**Supporting remote coaching**

- Setting, storing, and reviewing personal health- and sport-related goals; sharing them with clinicians for feasibility assessment, feedback, and personalization
- Tailored content, including clinically validated, personalized training schemes tailored to one’s sport, goals, current condition, and past performances
- Annotating clinical training suggestions with feedback grounded in personal experiences
- Receiving clinical feedback on current performance (eg, through annotations on remotely monitored exercise data)
- Multimedia, digital information resources about athletic patients with heart problems and how to deal with exercising while having a cardiac condition

**Supporting remote consultations**

- Periodic clinical check-ins on exercise management (eg, updating knowledge on the exercise limits or exercise-related symptoms) through synchronous or asynchronous channels, such as video calls or online surveys
- e-Consultation functionality allowing users to ask specific questions to appropriate clinicians via chat functionalities

## Discussion

### Principal Findings

#### Overview

Recent developments in the design of cardiac eHealth have encouraged HCI practitioners and designers to account for the patients’ latent and hidden needs, desires, and life values [32,48]. A recent scoping review on acceptance and adherence factors of CTR interventions emphasizes that early involvement of users in the design of CTR technologies is essential for designing meaningful and efficient interventions [54]. Nonetheless, there has been a lack of HCI studies on acknowledging the needs and values of patient subpopulations in CTR [33,34,54]. In this study, we outlined design requirements for technology-centric CTR interventions that cater to the values of a distinctive cardiac population—*athletes with established CAD*. We used methods from VSD and uncovered 12 key values of this specific population supported by underlying needs. These values included “independence and confidence in one’s body” and “concise, actionable guidelines,” alongside technology features grouped into design requirements addressing these values. In the following sections, we discuss the implications for the value-oriented design of CTR technologies in the context of the athletic population. We first situate our findings in the context of existing CTR research for general populations and then explore how alignments and conflicts among values can inform the design process. We discuss design guidelines based on the connections inferred between athlete values and the identified design requirements.

#### CTR for Athletic Populations Versus General Patient Groups

On the basis of our findings and previous, related work, the core experience of being a patient with a cardiac condition is fundamentally the same for both athletes and general populations. However, the key difference is that athletes have distinct attitudes toward physical activity, and their identity and discipline around it shape their expectations of care, particularly regarding physical activity training, the main component of CR. As a result, while there are similarities, CTR technologies should be designed differently for athletes compared to general populations. For example, general populations typically track any physical activity, such as steps (eg, MI-Pace [66]) or standardized exercises (eg, recommended aerobic exercises for patients with CVD [62]), whereas athletes prefer sport-specific tracking and feedback (eg, for cycling, running, or weight lifting). This difference can influence monitoring system design as our participants preferred relying on their own high-performance wearables, which they were trained to use and were of better quality than standard hospital-issued devices, whereas CTR systems for general populations are designed to offer patients standard monitoring devices (eg, Fitbit [62,76], Mio Alpha and ActiGraph [64], smartphone apps [30], and Philips health watch [66]). This can also affect personal insight platforms and clinician decision support systems, shifting from generic goals such as 10,000 steps per day (eg, shared care platform [94] and HeartPortal [74]) or 150 activity minutes per week [62] to sport-specific milestones based on frequency, distance, HR zones, and perceived effort.

Some needs remain the same, such as clear technical instructions and technical support for CTR platforms (eg, the MedFit app [62]) and seamless, continuous data transmission (eg, SmartCare-CAD [64]). However, graphical data representations alongside exercise limitations are particularly relevant for athletes as they need to moderate activity rather than increase it. Their familiarity with data tracking can make them more critical of inaccuracies and more confident in interpreting data, influencing their preference for precise alarm systems. In contrast, general populations may feel overwhelmed by excessive or highly technical data [4].

While data-focused discussions with clinicians are valuable for all patients [60,66], athletes require highly personalized exercise recommendations based on personal metrics, sport-specific testing, and clinical cardiology expertise. In addition, information resources designed for general populations (eg, ActiveHeart [76]) need to be enriched with athlete-specific content to prevent misinformation. The need for periodic consultations and clinician validation remains constant with athletes and general populations [64,66,76,94], but athletes may have more specialized questions, which could benefit from new communication methods such as prestructured forms, data annotations, or digital messaging for tailored interactions. We discuss the specific implications for athletic CTR technologies in the following subsections.

### Design Implications for CTR Technologies Supporting Athletes

#### Value Contradictions, Tensions, and Design Trade-Offs

Aligning with VSD theory, we observed value interrelations and tensions at various levels of human experience—among athletes, as well as between athletes and general populations of patients with cardiac conditions. The VSD framework acknowledges that human values do not exist in isolation [36]. Framing a design process to engage constructively in the interconnectedness of human values is a matter of design thinking and can be mediated through design trade-offs, value conflicts, or value tensions [78]. Such tensions, conflicts, and trade-offs have also been discussed in similar studies applying VSD methods to eHealth design [74,81]. The term *value tension* encourages the design of solutions that balance opposing values such that “the adjudication of the tension holds each value intact” [36]. In our case, most athletes valued data from wearable sensors to become better at sports or recalibrate internal feelings of exercise intensity, whereas other athletes (eg, P13 or P14) trusted the physical “feeling” enough and did not consider fitness trackers necessary. Therefore, one design outcome can be a wearable sensor system that offers insights into one’s performance and the meaning of data in the context of CAD after hospital dismissal, when athletes lack confidence in their bodies the most. Subsequently, tracking one’s training can become optional and periodical. On the other hand, a “design trade-off” conveys an approach in which designing for one value will diminish another value. For instance, designing a CTR technology that formalizes the role of a close family member and allows athletes to share personal data such as location and HR while exercising with them will come at the expense of the independence of other athletes who want to keep their experiences private. Offering this functionality for the safety and comfort of some athletes takes the form of a trade-off.

Our findings also align with previous research on the values of patients with cardiac conditions. The 11 values of patients with cardiac conditions as a foundation for eHealth development identified by Bente et al [50] overlap with the 12 identified values in our study (eg, “To have an overview of personal health data” overlaps with athletes’ value of “Health and performance quantification”). Nonetheless, there are also value conflicts. For instance, one key value of the population in the study by Bente et al [50] was “to be extrinsically motivated to accomplish goals or activities (related to health/lifestyle).” On the other hand, athletes valued a “goal and performance-oriented approach” to rehabilitation and were very independent in their health journey. The term *value conflict* proposes that, when values come into conflict, design resolutions can offer solutions responsive to both values. In the context of CTR individualization, one solution can be offering a standardized CTR platform that encompasses 2 different modules: one for patients who need behavior change (including information about the importance of exercise, motivational queues, and reminders) and one for athletes (including a more performance- and data-oriented interface comprising sport-specific goal setting, data overviews, and means to address sport-related questions).

While our results mostly show generalizations of common themes and requirements, designers should be aware that values and needs are expressed through the lens of individual experiences [79]. Two patients might have completely different rehabilitation experiences, whereas clinicians might benefit from solutions that patients do not necessarily need (eg, virtual assistants that can ease clinicians’ workload but are not perceived as trustworthy by athletes).

#### Connecting CTR Technology Requirements With Athlete Values

##### Preference for Hybrid CTR and Continuity of Care

**Figure 5:**
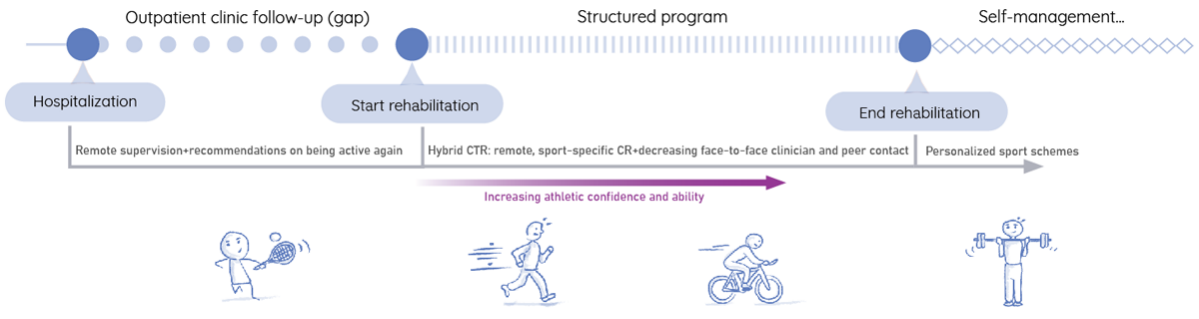
For athletes, cardiac telerehabilitation (CTR) provides chances to train in their sport at home before and during rehabilitation under remote supervision and receive guidance after the program ends. CR: cardiac rehabilitation.

Our research revealed a preference for a hybrid CTR intervention incorporating human-to-human contact for peer bonding and clinical consultations. This preference was particularly pronounced during the posthospitalization and early program stages, when there is a need for human interaction, emotional support, and sharing. Our findings also indicate a need for activity supervision and recommendations that can help regain activity after hospitalization, before the program starts. Similarly, previous research on general cardiac populations reveals that exposure to other patients can evoke feelings of safety, comfort, and camaraderie [61,95] and that support is needed in the posthospitalization gap [68]. However, athletes quickly build confidence, and their need for human contact can decrease fast after the start of the program. Peer support was seen as “nice to have” but not necessary, whereas athletes shifted their priorities toward regaining their physical condition. Although previous research on general populations suggests introducing technology later in the rehabilitation process as participants transition from program completion to self-management [11,95], our study indicates that athletes are prepared to become independent sooner. This readiness arises as soon as they regain physical strength and gain clarity regarding their new limitations, typically during the transition between the posthospitalization stage and the midpoint of the program. Our results indicate that, while the need for supervision may decrease significantly after the program ends, some participants still expressed a need for structured training plans to continue their progress (Figure 5). In the following subsections, we discuss the identified categories of CTR technology requirements that surfaced from our study and their connection to athlete values.

##### Supporting Remote Monitoring

Our participants expressed attitudes similar to those found in research on recreational athletes—sports are integral to athletes’ identity, well-being, and lifestyle [47]. They resisted the notion of being confined to a monotonous, standardized hospital-based program; however, their perception of safe sporting activities next to new physical limitations became distorted following the diagnosis. While athletes prioritized independence in their health journey, they still valued dependable clinical support significantly. This included receiving remote clinical monitoring of their sporting activities outside the hospital as well as biophysical measurements, ensuring ongoing clinical oversight. While current telemonitoring in CTR focuses on patient adherence to exercise targets and symptom management [62,94], for athletes, its purpose would shift toward preventing overexertion and tailoring future training programs based on their heart’s capabilities and symptoms. Moreover, current telemonitoring relies on step counters, distance, or data indicating the presence of physical activity [54,61]. However, a more nuanced approach is needed for athletes, incorporating detailed sport-specific parameters such as type of sport, intensity minutes, HR zones, and HR recovery, similar to insights found in sports apps such as Strava or JOIN. Furthermore, while regular subjective data gathering, such as diaries, was not considered essential, some athletes expressed a need for documenting anxieties, particularly those related to sports, symptoms, or specific workout instances. Such documentation could provide a personalized touch and reassurance. This could be facilitated through open methods of annotating sensor data, as recently discussed by Tadas et al [11] or Akinsiku et al [96]. As already documented, athletes are accustomed to self-monitoring—they already have preferences and high expectations related to reliable monitoring systems [46,47]. Our findings show that both athletes and HCPs prefer using the athletes’ wearable sensors because of familiarity, perceived device quality, and accuracy. Despite potential challenges such as data inconsistencies and integration issues, leveraging existing resources can save costs and offer valuable historical insights predating diagnosis.

##### Supporting Human-Data Interaction

Previous literature has underlined the trained capacity of recreational athletes to be aware of signals sent by their bodies, using data from wearable sensors (mostly HR) to recalibrate internal feelings of embodied experience [46,47]. Our research confirms these findings, with most participants using wearable sensors during training for self-reflection and internal recalibration. Some athletes even asserted their deep trust in their body’s response to exertion, suggesting that they relied more on familiarity than data. However, this equilibrium shifted after hospitalization, when even basic activities became challenging. Accepting one’s limitations and adapting to new boundaries is crucial. Athletes undergo a recalibration process by relearning how data, symptoms, and bodily sensations relate to and mutually influence one another. Graphical representations of physical and athletic progress next to clinical validation can facilitate the creation of “in-the-moment knowledge” [48] and facilitate the recalibration process. Access to clinical tests (eg, exercise stress tests), data representations incorporating clinical limitations such as maximum allowed HR, real-time notifications for exceeding limitations during exercise, and alerts for clinicians and athletes regarding data anomalies can facilitate accepting one’s condition. An example of real-time training feedback based on optimal training zones is present in the work by Geurts et al [97]. These enhancements become indispensable as wearable data evolve beyond mere performance metrics [46] following a diagnosis. However, some athletes expressed skepticism regarding the accuracy and reliability of the data, recognizing that their sensors were not medical devices [98]. As a result, they sought clinical validation and aspired to engage in data-driven discussions with their clinicians even during brief online consultations. Therefore, a CTR system could streamline the process by enabling easy access to data from specific periods during these e-consultations. In addition, in contrast to findings of general cardiac populations being open to sharing collected data with peers [11], our participants found sharing wearable sensor data with peers irrelevant both before and after their diagnosis. Previous studies similarly indicate that the social value of recreational athletes’ practice is not found through online communities or peer data sharing [47,49]. Finally, while the family may play a less active role in an athlete’s physical recovery, they are significantly involved in mediating anxiety and emotional healing. Designers must consider the social dynamics inherent in a CTR system and its monitored data, as discussed by Andersen et al [45]. They can address family safety concerns and partner anxiety through location sharing and negotiation mechanisms akin to features in apps such as Strava or Apple Fitness+. To streamline and enhance conversations and reflections about data, designers should implement strategies that cater to low levels of data literacy and effectively communicate progress or trends, as evidenced by previous research [99,100].

##### Supporting Remote Coaching

A key finding of our study highlights athletes’ perception of inadequate care in colocated CR as their exercise needs were overlooked due to the assumption that they were “fitter” than other patients. Unlike in general populations in which low motivation contributes to poor program adherence [101], athletes’ enjoyment and willingness to attend were hindered by not being guided to exercise at the desired intensity. As patients with cardiac conditions are keen on returning to normality [95], athletes are eager to reclaim their peak physical condition. In their pursuit, they merge health and athletic objectives, such as aiming to increase their personal best timing to eventually participate in a half-marathon. While goal setting is addressed in current CTR designs, nonetheless, clinicians are the main initiators or coordinators of physical activity goals [76]. Catering to athletes’ independence and autonomy, athlete-centered CTR interventions should empower athletes to establish their sport-specific performance goals, incorporating motivators and metrics (eg, desired running distance) while facilitating clinician feedback and adjustments. In line with our findings, recent research on consumer wearable systems for sports highlights the significance of incorporating sport-specific goals and recommendations as a crucial design enhancement [102].

To support values such as “To be extrinsically motivated to accomplish goals” [50], current CTR interventions focus on enhancing program engagement through persuasive strategies (eg, multimedia content [25] or collaborative gamified experiences [65]). In contrast, athletes do not require external motivators such as rewards or gamification but rather need facilitators such as personalized, sport-based training content with adapting intensity that can be static (eg, textual) but rich in clinically valid information such as intensity specificity and limitations. The CTR platform developed by Sankaran et al [27] provides a notable example of detailed visualizations depicting exercise prescriptions and progress, which could be customized for the athletic population. A crucial design feature could also involve aligning clinically suggested exercise schemes with data from the performed workout for comparison, enabling athletes to offer feedback to clinicians based on their personal experiences with the suggested exercises. Finally, to ensure a clinically validated understanding of athletes’ specific situation, CTR platforms should offer personalized information resources on exercising with established CAD. Athletes currently rely on inconsistent and potentially unsafe online sources for safe exercise.

##### Supporting Remote Consultations

Athletes experience a high level of anxiety after hospitalization, and receiving care with a personal touch can alleviate mental struggles. These values can be addressed by facilitating person-to-person care tailored to the patients’ values, beliefs, and life goals [32]. As athletes regain confidence, reacquaint themselves with their limits, and recalibrate their internal sense of “body safety,” their inquiries to clinicians become more specific and briefer (eg, regarding symptoms, medication, or limitations). Unfortunately, some athletes struggled with alleviating their anxieties in the program and found relationships with clinicians cold or inaccessible. To maintain personal connectivity with the health care system and avoid depersonalization of remotely gathered data [103], CTR technologies for athletes ought to incorporate periodic “check-in points”—online consultations via synchronous or asynchronous methods. Some participants proposed periodic surveys concerning symptoms or physical recovery. In addition, athletes occasionally expressed the need to ask situational questions through chat functionalities or features akin to e-consultations. Such findings align with current HCI investigations of cardiac telehealth that emphasize the benefits of harnessing HCP-patient relationships through remote care [31,50,61,94].

### Suggestions for Future Work and Limitations

Considering the significant individual and public health challenges posed by CVDs, along with increasing medical knowledge on the risks of physical exertion in recreational athletes with cardiac conditions, CTR interventions must move beyond one-size-fits-all approaches toward highly personalized, data-driven, and athlete-centered rehabilitation experiences that empower patients to safely return to peak performance. Future research should focus on translating the identified design implications and requirements into CTR solutions tailored for athletes followed by iterative validation and implementation with key stakeholders, including athletes, caregivers, and clinicians. Operationalizing VSD in the design of complex CTR interventions will take more than making connections between values and feature requirements [32]. Future work can investigate the translation of value-sensitive requirements to CTR design and validation by using, for instance, the value hierarchies by Van de Poel [104] and validating value-sensitive CTR in actual effectiveness trials using methods such as the Multiphase Optimization Strategy [105].

Our findings provide a broad understanding of the problem at hand because of the nature of the qualitative methods. However, our study had some limitations. All participants were Dutch either being treated or working at the same hospital. We especially acknowledge the gender imbalance in our recruited patients (only 1/15, 7% were women) as the underrepresentation of women in CR research is a documented, fundamental problem [106]. One constraint was the inclusion criterion, limiting enrollment to participants who had completed the CR program within 1 year before the study’s start, which restricted opportunities to include more women. Nonetheless, we recognize that this might be a systemic problem related to women’s diagnosis, referral patterns, or adherence to CR programs [107,108]. These considerations may limit the generalizability to more diverse groups, such as women or athletes being treated in other cultures, countries, or jurisdictions. Future research should investigate the values and needs of such groups.

We included only patients who exercised at least 4 hours per week before diagnosis, but our findings may also apply to less athletic but active individuals. While athletes have distinct values and needs compared to general cardiac populations, some preferences overlap, warranting further research on their generalizability to a larger sample of active patients. In addition, the values of athletes with established CAD should be examined alongside those of healthy recreational athletes to better understand implications for fitness- and health-tracking technologies for athletes with cardiac conditions.

CTR is a complex combination of face-to-face and remote services, requiring consideration of technological (eg, data representations), service (eg, consultations), and functional (eg, sensors vs surveys) features. Design choices in this study were shaped by the interviewed population, making some requirements broad and exploratory. Future research should explore athletes’ preferences for specific CTR functionalities (eg, alarms for red flags in the data). In addition, accessibility should be a key consideration (eg, a participant with a hearing disability emphasized the need for reliable video streaming to support lip reading, highlighting the importance of inclusive design in CTR interventions).

Finally, within HCI literature, there is criticism of ethnographic studies being evaluated on the implications they can have for design—this can give rise to the idea that design is the end point of the research [109,110]. Our study indicates opportunities for reorganizing and leveraging existing CR services for athlete rehabilitation that could be explored further in future research. While beyond this study’s scope, discussions on implications for policy makers and health care institutions warrant attention in future work.

### Conclusions

This study contributes to understanding how CTR technologies can address the needs and values of athletes with established CAD. Our results show that, for the independent, highly active athletic population, CTR interventions should be more focused on *remote performance tracking*, *clear data reports*, and *communication channels that enable shared decision-making between athletes and clinicians*. We propose design considerations for value-oriented CTR technologies aimed at the athletic population and encourage designers to account for the contrasting values and needs of different stakeholders in the design process. From a practical standpoint, these findings can inform the development of CTR platforms that better align with athletes’ expectations by integrating performance feedback, sport-specific metrics, and adaptive supervision. Clinicians and technologists can collaborate to leverage these insights and personalize remote rehabilitation care models that gradually shift from structured guidance to independent athlete-led monitoring. In addition, health care institutions can use these results to refine hybrid rehabilitation programs, balancing digital autonomy with strategic in-person evaluations to optimize adherence and safety. Future implementations should prioritize accessibility, interoperability with wearable devices, and customizable consultation models to enhance athlete-centered care in CTR.

## Appendix

Multimedia Appendix 1 Interview scripts for athletes and health care professionals.

Multimedia Appendix 2 The cards used in the card-sorting focus groups.

Multimedia Appendix 3 The codebook developed after analyzing the interview results and example quotes.

Multimedia Appendix 4 Identified athlete values with paired needs and example quotes from the interview phase.

Multimedia Appendix 5 The card-sorting activity results—all cards sorted from most needed to least needed.

Multimedia Appendix 6 Derived cardiac telerehabilitation technology requirements paired with athlete values, where “x” indicates a match between a feature and a value; the cards used to derive the requirements; and example notes or observations made during the card-sorting activity.

## Abbreviations

CAD: coronary artery disease
CR: cardiac rehabilitation
CTR: cardiac telerehabilitation
CVD: cardiovascular disease
FG: focus group
HCD: human-centered design
HCI: human-computer interaction
HCP: health care professional
HR: heart rate
PHR: participatory health research
VSD: value-sensitive design

## References

[1] Wilkins E, Wilson L, Wickramasinghe K, Bhatnagar P, Leal J, Luengo-Fernandez R, Burns R, Rayner M, Townsend N (2017). European Cardiovascular Disease Statistics 2017.

[2] Pelliccia A, Sharma S, Gati S, Bäck Maria, Börjesson Mats, Caselli S, Collet JP, Corrado D, Drezner JA, Halle M, Hansen D, Heidbuchel H, Myers J, Niebauer J, Papadakis M, Piepoli MF, Prescott E, Roos-Hesselink JW, Graham Stuart A, Taylor RS, Thompson PD, Tiberi M, Vanhees L, Wilhelm M, ESC Scientific Document Group (2021). 2020 ESC Guidelines on sports cardiology and exercise in patients with cardiovascular disease. Eur Heart J.

[3] Anderson L, Oldridge N, Thompson DR, Zwisler AD, Rees K, Martin N, Taylor RS (2016). Exercise-based cardiac rehabilitation for coronary heart disease: Cochrane systematic review and meta-analysis. J Am Coll Cardiol.

[4] Kondratova I, Fournier H (2022). Virtual cardiac rehabilitation in a pandemic scenario: a review of HCI design features, user acceptance and barriers. Proceedings of the 8th International Conference on Human Aspects of IT for the Aged Population.

[5] Peretti A, Amenta F, Tayebati SK, Nittari G, Mahdi SS (2017). Telerehabilitation: review of the state-of-the-art and areas of application. JMIR Rehabil Assist Technol.

[6] Nilsson U, Öberg B, Bäck M (2023). Patients' perceptions of exercise-based cardiac telerehabilitation after a myocardial infarction-a qualitative study. Int J Environ Res Public Health.

[7] Davis MM, Freeman M, Kaye J, Vuckovic N, Buckley DI (2014). A systematic review of clinician and staff views on the acceptability of incorporating remote monitoring technology into primary care. Telemed J E Health.

[8] Morimoto Y, Takahashi T, Sawa R, Saitoh M, Morisawa T, Kagiyama N, Kasai T, Dinesen B, Hollingdal M, Refsgaard J, Daida H (2022). Web portals for patients with chronic diseases: scoping review of the functional features and theoretical frameworks of telerehabilitation platforms. J Med Internet Res.

[9] Munteanu C, Axtell B, Rafih H, Liaqat A, Aly Y, Agnew J, Mitchell OS (2019). Designing for older adults: overcoming barriers to a supportive, safe, and healthy retirement. The Disruptive Impact of FinTech on Retirement Systems.

[10] Tsai TH, Lin WY, Chang YS, Chang PC, Lee MY (2020). Technology anxiety and resistance to change behavioral study of a wearable cardiac warming system using an extended TAM for older adults. PLoS One.

[11] Tadas S, Dickson J, Coyle D (2023). Using patient-generated data to support cardiac rehabilitation and the transition to self-care. Proceedings of the 2023 CHI Conference on Human Factors in Computing Systems.

[12] Churchill TW, Baggish AL (2020). Cardiovascular care of masters athletes. J Cardiovasc Transl Res.

[13] Aengevaeren VL, Mosterd A, Braber TL, Prakken NH, Doevendans PA, Grobbee DE, Thompson PD, Eijsvogels TM, Velthuis BK (2017). Relationship between lifelong exercise volume and coronary atherosclerosis in athletes. Circulation.

[14] Mittleman MA, Maclure M, Tofler GH, Sherwood JB, Goldberg RJ, Muller JE (1993). Triggering of acute myocardial infarction by heavy physical exertion. Protection against triggering by regular exertion. Determinants of Myocardial Infarction Onset Study Investigators. N Engl J Med.

[15] Čulić V, Alturki A, Vio R, Proietti R, Jerončić A (2023). Acute myocardial infarction triggered by physical exertion: a systematic review and meta-analysis. Eur J Prev Cardiol.

[16] Claessen G, Eijsvogels TM, Albert CM, Baggish AL, Levine BD, Marijon E, Michos ED, La Gerche A (2025). Coronary atherosclerosis in athletes: emerging concepts and preventive strategies. Eur Heart J.

[17] McKinney J, Velghe J, Fee J, Isserow S, Drezner JA (2019). Defining athletes and exercisers. Am J Cardiol.

[18] Kinast B, Lutz M, Schreiweis B (2021). Telemonitoring of real-world health data in cardiology: a systematic review. Int J Environ Res Public Health.

[19] Kakria P, Tripathi NK, Kitipawang P (2015). A real-time health monitoring system for remote cardiac patients using smartphone and wearable sensors. Int J Telemed Appl.

[20] Worringham C, Rojek A, Stewart I (2011). Development and feasibility of a smartphone, ECG and GPS based system for remotely monitoring exercise in cardiac rehabilitation. PLoS One.

[21] De Cannière H, Corradi F, Smeets CJ, Schoutteten M, Varon C, Van Hoof C, Van Huffel S, Groenendaal W, Vandervoort P (2020). Wearable monitoring and interpretable machine learning can objectively track progression in patients during cardiac rehabilitation. Sensors (Basel).

[22] Thijs I, Fresiello L, Oosterlinck W, Sinnaeve P, Rega F (2019). Assessment of physical activity by wearable technology during rehabilitation after cardiac surgery: explorative prospective monocentric observational cohort study. JMIR Mhealth Uhealth.

[23] Pavy B, Darchis J, Merle E, Caillon M (2016). [Cardiac rehabilitation in "sports" patients]. Ann Cardiol Angeiol (Paris).

[24] Borjesson M, Dellborg M, Niebauer J, LaGerche A, Schmied C, Solberg EE, Halle M, Adami E, Biffi A, Carré F, Caselli S, Papadakis M, Pressler A, Rasmusen H, Serratosa L, Sharma S, van Buuren F, Pelliccia A (2019). Recommendations for participation in leisure time or competitive sports in athletes-patients with coronary artery disease: a position statement from the sports cardiology section of the European Association of Preventive Cardiology (EAPC). Eur Heart J.

[25] Sankaran S, Luyten K, Hansen D, Dendale P, Coninx K (2019). Enhancing patient motivation through intelligibility in cardiac tele-rehabilitation. Interact Comput.

[26] Sankaran S, Frederix I, Haesen M, Dendale P, Luyten K, Coninx K (2016). A grounded approach for applying behavior change techniques in mobile cardiac tele-rehabilitation. Proceedings of the 9th ACM International Conference on PErvasive Technologies Related to Assistive Environments.

[27] Sankaran S, Luyten K, Hansen D, Coninx K (2018). Have you met your METs?: enhancing patient motivation to achieve physical activity targets in cardiac tele-rehabilitation. Proceedings of the 32nd International BCS Human Computer Interaction Conference.

[28] Quazi S, Malik JA (2022). A systematic review of personalized health applications through human-computer interactions (HCI) on cardiovascular health optimization. J Cardiovasc Dev Dis.

[29] Brouwers RW, Brini A, Kuijpers RW, Kraal JJ, Kemps HM (2022). Predictors of non-participation in a cardiac telerehabilitation programme: a prospective analysis. Eur Heart J Digit Health.

[30] Falter M, Scherrenberg M, Dendale P (2020). Digital health in cardiac rehabilitation and secondary prevention: a search for the ideal tool. Sensors (Basel).

[31] Beleigoli A, Champion S, Tirimacco R, Nesbitt K, Tideman P, Clark RA (2021). A co-designed telehealth-based model of care to improve attendance and completion to cardiac rehabilitation of rural and remote Australians: the Country Heart Attack Prevention (CHAP) project. J Telemed Telecare.

[32] Cruz-Martínez RR, Wentzel J, Bente BE, Sanderman R, van Gemert-Pijnen JE (2021). Toward the value sensitive design of eHealth technologies to support self-management of cardiovascular diseases: content analysis. JMIR Cardio.

[33] Hwang R, Gane EM, Morris NR (2023). No transport? No worries! Cardiac telerehabilitation is a feasible and effective alternative to centre-based programs. Heart Fail Rev.

[34] Brouwers RW, van Exel HJ, van Hal JM, Jorstad HT, de Kluiver EP, Kraaijenhagen RA, Kuijpers PM, van der Linde MR, Spee RF, Sunamura M, Uszko-Lencer NH, Vromen T, Wittekoek ME, Kemps HM (2020). Cardiac telerehabilitation as an alternative to centre-based cardiac rehabilitation. Neth Heart J.

[35] Friedman B, Kahn PH Jr, Borning A, Huldtgren A, Doorn N, Schuurbiers D, van de Poel I, Gorman M (2013). Value sensitive design and information systems. Early Engagement and New Technologies: Opening Up the Laboratory.

[36] Friedman B, Hendry DG (2019). Value Sensitive Design: Shaping Technology with Moral Imagination.

[37] Cruz-Martínez RR, Wentzel J, Asbjørnsen RA, Noort PD, van Niekerk JM, Sanderman R, van Gemert-Pijnen JE (2020). Supporting self-management of cardiovascular diseases through remote monitoring technologies: metaethnography review of frameworks, models, and theories used in research and development. J Med Internet Res.

[38] Song Y, Ren C, Liu P, Tao L, Zhao W, Gao W (2020). Effect of smartphone-based telemonitored exercise rehabilitation among patients with coronary heart disease. J Cardiovasc Transl Res.

[39] Anderson L, Sharp GA, Norton RJ, Dalal H, Dean SG, Jolly K, Cowie A, Zawada A, Taylor RS (2017). Home-based versus centre-based cardiac rehabilitation. Cochrane Database Syst Rev.

[40] Maddison R, Rawstorn JC, Stewart RA, Benatar J, Whittaker R, Rolleston A, Jiang Y, Gao L, Moodie M, Warren I, Meads A, Gant N (2019). Effects and costs of real-time cardiac telerehabilitation: randomised controlled non-inferiority trial. Heart.

[41] Snoek JA, Prescott EI, van der Velde AE, Eijsvogels TM, Mikkelsen N, Prins LF, Bruins W, Meindersma E, González-Juanatey JR, Peña-Gil C, González-Salvado V, Moatemri F, Iliou MC, Marcin T, Eser P, Wilhelm M, Van't Hof AW, de Kluiver EP (2021). Effectiveness of home-based mobile guided cardiac rehabilitation as alternative strategy for nonparticipation in clinic-based cardiac rehabilitation among elderly patients in Europe: a randomized clinical trial. JAMA Cardiol.

[42] Schmid J, Adams J, Cheng D (2009). Cardiac rehabilitation of a 77-year-old male runner: consideration of the athlete, not the age. Proc (Bayl Univ Med Cent).

[43] Hathorn B, Rodgers L (2022). Cardiac rehabilitation testing of a high-intensity performance athlete firefighter after myocardial infarction, placement of stents and an implantable cardioverter-defibrillator. Proc (Bayl Univ Med Cent).

[44] Weber N, Weber A, Carbone P, Lawrence A, Bilbrey T, Schussler JM, Adams J (2018). High-intensity, sport-specific cardiac rehabilitation training of a 22-year-old competitive cyclist after spontaneous coronary artery dissection. Proc (Bayl Univ Med Cent).

[45] Andersen TO, Langstrup H, Lomborg S (2020). Experiences with wearable activity data during self-care by chronic heart patients: qualitative study. J Med Internet Res.

[46] Pandey M, Nebeling M, Park SY, Oney S (2019). Exploring tracking needs and practices of recreational athletes. Proceedings of the EAI International Conference on Pervasive Computing Technologies for Healthcare - Demos and Posters.

[47] Tholander J, Nylander s (2015). Snot, sweat, pain, mud, and snow: performance and experience in the use of sports watches. Proceedings of the 33rd Annual ACM Conference on Human Factors in Computing Systems.

[48] Tadas S, Coyle D (2020). Barriers to and facilitators of technology in cardiac rehabilitation and self-management: systematic qualitative grounded theory review. J Med Internet Res.

[49] Dionigi RA, Fraser-Thomas J, Logan J (2012). The nature of family influences on sport participation in Masters athletes. Ann Leis Res.

[50] Bente BE, Wentzel J, Groeneveld RG, IJzerman RV, de Buisonjé DR, Breeman LD, Janssen VR, Kraaijenhagen R, Pieterse ME, Evers AW, van Gemert-Pijnen JE (2021). Values of importance to patients with cardiovascular disease as a foundation for eHealth design and evaluation: mixed methods study. JMIR Cardio.

[51] Hartrevalidatie. Hartstichting.

[52] Corrà U, Piepoli MF, Carré F, Heuschmann P, Hoffmann U, Verschuren M, Halcox J, Giannuzzi P, Saner H, Wood D, Piepoli MF, Corrà U, Benzer W, Bjarnason-Wehrens B, Dendale P, Gaita D, McGee H, Mendes M, Niebauer J, Zwisler AD, Schmid JP, European Association of Cardiovascular PreventionRehabilitation Committee for Science Guidelines, EACPR, Document Reviewers (2010). Secondary prevention through cardiac rehabilitation: physical activity counselling and exercise training: key components of the position paper from the Cardiac Rehabilitation Section of the European Association of Cardiovascular Prevention and Rehabilitation. Eur Heart J.

[53] Cramer SC, Grotta JC, Albers GW, Broderick JP, Kasner SE, Lo EH, Mendelow AD, Sacco RL, Wong LK (2015). Interventions to improve recovery after stroke. Stroke: Pathophysiology, Diagnosis, and Management.

[54] Ramachandran HJ, Jiang Y, Teo JY, Yeo TJ, Wang W (2022). Technology acceptance of home-based cardiac telerehabilitation programs in patients with coronary heart disease: systematic scoping review. J Med Internet Res.

[55] Burridge JH, Lee AC, Turk R, Stokes M, Whitall J, Vaidyanathan R, Clatworthy P, Hughes AM, Meagher C, Franco E, Yardley L (2017). Telehealth, wearable sensors, and the internet: will they improve stroke outcomes through increased intensity of therapy, motivation, and adherence to rehabilitation programs?. J Neurol Phys Ther.

[56] Conroy SS, Harcum S, Keldsen L, Bever CT Jr (2020). Novel use of existing technology: a preliminary study of patient portal use for telerehabilitation. J Telemed Telecare.

[57] Afyouni I, Einea A, Murad A (2019). Towards an adaptive gaming framework for TeleRehabilitation. Proceedings of the 12th ACM International Conference on PErvasive Technologies Related to Assistive Environmen.

[58] Dinesen B, Dittmann L, Gade JD, Jørgensen CK, Hollingdal M, Leth S, Melholt C, Spindler H, Refsgaard J (2019). "Future patient" telerehabilitation for patients with heart failure: protocol for a randomized controlled trial. JMIR Res Protoc.

[59] Melholt C, Joensson K, Spindler H, Hansen J, Andreasen JJ, Nielsen G, Noergaard A, Tracey A, Thorup C, Kringelholt R, Dinesen BI (2018). Cardiac patients' experiences with a telerehabilitation web portal: implications for eHealth literacy. Patient Educ Couns.

[60] Kjærup M, Kouzeli S, Skov MB, Kjeldskov J, Skov CS, Søgaard P (2018). Diagnostic agents: collaborative interpretation for cardiac patients at home. Proceedings of the Extended Abstracts of the 2018 CHI Conference on Human Factors in Computing Systems.

[61] Clark AM, Sousa BJ, Ski CF, Redfern J, Neubeck L, Allana S, Peart A, MacDougall D, Thompson DR (2023). Main mechanisms of remote monitoring programs for cardiac rehabilitation and secondary prevention: a systematic review. J Cardiopulm Rehabil Prev.

[62] Duff O, Walsh D, Malone S, McDermott L, Furlong B, O'Connor N, Moran K, Woods C (2018). MedFit app, a behavior-changing, theoretically informed mobile app for patient self-management of cardiovascular disease: user-centered development. JMIR Form Res.

[63] Athilingam P, Labrador MA, Remo EF, Mack L, San Juan AB, Elliott AF (2016). Features and usability assessment of a patient-centered mobile application (HeartMapp) for self-management of heart failure. Appl Nurs Res.

[64] Brouwers RW, Kraal JJ, Traa SC, Spee RF, Oostveen LM, Kemps HM (2017). Effects of cardiac telerehabilitation in patients with coronary artery disease using a personalised patient-centred web application: protocol for the SmartCare-CAD randomised controlled trial. BMC Cardiovasc Disord.

[65] Dithmer M, Rasmussen JO, Grönvall E, Spindler H, Hansen J, Nielsen G, Sørensen SB, Dinesen B (2016). "The heart game": using gamification as part of a telerehabilitation program for heart patients. Games Health J.

[66] Ding EY, Erskine N, Stut W, McManus DD, Peterson A, Wang Z, Escobar Valle J, Albuquerque D, Alonso A, Botkin NF, Pack QR, McManus DD (2021). MI-PACE home-based cardiac telerehabilitation program for heart attack survivors: usability study. JMIR Hum Factors.

[67] Hallberg I, Ranerup A, Bengtsson U, Kjellgren K (2018). Experiences, expectations and challenges of an interactive mobile phone-based system to support self-management of hypertension: patients’ and professionals’ perspectives. Patient Prefer Adherence.

[68] Rawstorn JC, Gant N, Rolleston A, Whittaker R, Stewart R, Benatar J, Warren I, Meads A, Jiang Y, Maddison R (2018). End users want alternative intervention delivery models: usability and acceptability of the REMOTE-CR exercise-based cardiac telerehabilitation program. Arch Phys Med Rehabil.

[69] Holeman I, Kane D (2019). Human-centered design for global health equity. Inf Technol Dev.

[70] Min A, Miller WR, Rocha LM, Börner K, Correia RB, Shih PC (2023). Understanding contexts and challenges of information management for epilepsy care. Proceedings of the 2023 CHI Conference on Human Factors in Computing Systems.

[71] Cha YJ, Wou A, Saxena A, Lee J, Newman MW, Park SY (2023). It’s like an educated guessing game: parents’ strategies for collaborative diabetes management with their children. Proceedings of the 2023 CHI Conference on Human Factors in Computing Systems.

[72] Sepehri K, Holsti L, Niasati S, Chan V, Maclean KE (2023). Beyond the bulging binder: family-centered design of a digital health information management system for caregivers of children living with health complexity. Proceedings of the 2023 CHI Conference on Human Factors in Computing Systems.

[73] Bhat KS, Hall AK, Kuo T, Kumar N (2023). "We are half-doctors": family caregivers as boundary actors in chronic disease management. Proc ACM Hum Comput Interact.

[74] Joensson K, Melholt C, Hansen J, Leth S, Spindler H, Olsen MV, Dinesen B (2019). Listening to the patients: using participatory design in the development of a cardiac telerehabilitation web portal. Mhealth.

[75] Khanshan A, Giling L, Markopoulos P, Van Gorp P (2023). A case study of data-enabled design for cardiac telemonitoring. Proceedings of the European Conference on Cognitive Ergonomics 2023.

[76] Dinesen B, Nielsen G, Andreasen JJ, Spindler H (2019). Integration of rehabilitation activities into everyday life through telerehabilitation: qualitative study of cardiac patients and their partners. J Med Internet Res.

[77] Friedman B, Kahn PH Jr, Borning a, Himma KE, Tavani HT (2006). Value sensitive design and information systems. The Handbook of Information and Computer Ethics.

[78] Friedman B, Hendry DG, Borning A (2017). A survey of value sensitive design methods. Found Trends Hum Comput Interact.

[79] van Gemert-Pijnen JE, Nijland N, van Limburg M, Ossebaard HC, Kelders SM, Eysenbach G, Seydel ER (2011). A holistic framework to improve the uptake and impact of eHealth technologies. J Med Internet Res.

[80] Cenci A, Ilskov SJ, Andersen NS, Chiarandini M (2023). The participatory value-sensitive design (VSD) of a mHealth app targeting citizens with dementia in a Danish municipality. AI Ethics.

[81] Asbjørnsen RA, Wentzel J, Smedsrød ML, Hjelmesæth J, Clark MM, Solberg Nes L, Van Gemert-Pijnen JE (2020). Identifying persuasive design principles and behavior change techniques supporting end user values and needs in eHealth interventions for long-term weight loss maintenance: qualitative study. J Med Internet Res.

[82] Giacomin J (2015). What is human centred design?. Design J.

[83] Wright MT (2015). What is participatory health research? A position paper of the international collaboration for participatory health research. Eur J Public Health.

[84] Knoche H, Abdul-Rahman A, Clark L, Curcin V, Huo Z, Iwaya LH, Lemon O, Mikulík R, Neate T, Skovfoged MM, Verdezoto N, Wilson S, Ziadeh H (2023). Identifying challenges and opportunities for intelligent data-driven health interfaces to support ongoing care. Proceedings of the Extended Abstracts of the 2023 CHI Conference on Human Factors in Computing Systems.

[85] van den Hoven J, Vermaas PE, van de Poel I (2015). Handbook of Ethics, Values, and Technological Design: Sources, Theory, Values and Application Domains.

[86] Mantzana V, Themistocleous M, Irani Z, Morabito V (2017). Identifying healthcare actors involved in the adoption of information systems. Eur J Inf Syst.

[87] Hennink M, Kaiser BN (2022). Sample sizes for saturation in qualitative research: a systematic review of empirical tests. Soc Sci Med.

[88] Squire CM, Giombi KC, Rupert DJ, Amoozegar J, Williams P (2024). Determining an appropriate sample size for qualitative interviews to achieve true and near code saturation: secondary analysis of data. J Med Internet Res.

[89] Saba MA, Goharpey S, Attarbashi Moghadam B, Salehi R, Nejatian M (2021). Correlation between the 6-min walk test and exercise tolerance test in cardiac rehabilitation after coronary artery bypass grafting: a cross-sectional study. Cardiol Ther.

[90] Kraal JJ, Peek N, Van den Akker-Van Marle ME, Kemps HM (2014). Effects of home-based training with telemonitoring guidance in low to moderate risk patients entering cardiac rehabilitation: short-term results of the FIT@Home study. Eur J Prev Cardiol.

[91] Razzouk R, Shute V (2012). What is design thinking and why is it important?. Rev Educ Res.

[92] Spencer D, Garrett JJ (2009). Card Sorting: Designing Usable Categories.

[93] Braun V, Clarke V (2006). Using thematic analysis in psychology. Qual Res Psychol.

[94] Dinesen B, Spindler H (2018). The use of telerehabilitation technologies for cardiac patients to improve rehabilitation activities and unify organizations: qualitative study. JMIR Rehabil Assist Technol.

[95] Tadas S, Pretorius C, Foster EJ, Gorely T, Leslie SJ, Coyle D (2021). Transitions in technology-mediated cardiac rehabilitation and self-management: qualitative study using the theoretical domains framework. JMIR Cardio.

[96] Akinsiku A, Avellino I, Graham Y, Mentis HM (2021). It’s not just the movement: experiential information needed for stroke telerehabilitation. Proceedings of the 2021 CHI Conference on Human Factors in Computing Systems.

[97] Geurts E, Haesen M, Dendale P, Luyten K, Coninx K (2016). Back on bike: the BoB mobile cycling app for secondary prevention in cardiac patients. Proceedings of the 18th International Conference on Human-Computer Interaction with Mobile Devices and Service.

[98] Andersen TO, Fritsch J, Matthiesen S (2023). Patient data work with consumer self-tracking: exploring affective and temporal dimensions in chronic self-care. Pervasive Computing Technologies for Healthcare.

[99] Gupta A, Heng T, Shaw C, Gromala D, Leese J, Li L (2020). Oh, I didn't do a good job: how objective data affects physiotherapist-patient conversations for arthritis patients. Proceedings of the 14th EAI International Conference on Pervasive Computing Technologies for Healthcare.

[100] Seals A, Pilloni G, Kim J, Sanchez R, Rizzo JR, Charvet L, Nov O, Dove G (2022). ‘Are they doing better in the clinic or at home?’: understanding clinicians’ needs when visualizing wearable sensor data used in remote gait assessments for people with multiple sclerosis. Proceedings of the 2022 CHI Conference on Human Factors in Computing Systems.

[101] Cohen Rodrigues TR, de Buisonjé DR, Keesman M, Reijnders T, van der Geer JE, Janssen VR, Kraaijenhagen RA, Atsma DE, Evers AW (2021). Facilitators of and barriers to lifestyle support and eHealth solutions: interview study among health care professionals working in cardiac care. J Med Internet Res.

[102] Mencarini E, Rapp A, Tirabeni L, Zancanaro M (2019). Designing wearable systems for sports: a review of trends and opportunities in human–computer interaction. IEEE Trans Hum Mach Syst.

[103] Primenko DV, Spevakov AG, Spevakova SV, Radionov A, Karandaev A (2020). Depersonalization of personal data in information systems. Advances in Automation.

[104] van de Poel I, Michelfelder D, McCarthy N, Goldberg D (2013). Translating values into design requirements. Philosophy and Engineering: Reflections on Practice, Principles and Process.

[105] Collins LM (2018). Optimization of Behavioral, Biobehavioral, and Biomedical Interventions: The Multiphase Optimization Strategy (MOST).

[106] Cugusi L, Mercuro G (2019). A systematic overview to quantify the gender imbalance in cardiovascular rehabilitation trials. Eur J Prev Cardiol.

[107] Kristofferzon ML, Löfmark R, Carlsson M (2007). Striving for balance in daily life: experiences of Swedish women and men shortly after a myocardial infarction. J Clin Nurs.

[108] Jackson L, Leclerc J, Erskine Y, Linden W (2005). Getting the most out of cardiac rehabilitation: a review of referral and adherence predictors. Heart.

[109] van Berkel N, Hornbæk K (2023). Implications of human-computer interaction research. Interactions.

[110] Dourish P (2006). Implications for design. Proceedings of the SIGCHI Conference on Human Factors in Computing Systems.

