# Supplementary material for: Design Requirements for Cardiac Telerehabilitation Technologies Supporting Athlete Values: Qualitative Interview Study
Source: JMIR Rehabil Assist Technol. 2025 Apr 17;12:e62986. doi: 10.2196/62986 (PMC12046260; doi:10.2196/62986)
Supplement: Multimedia Appendix 1 [file rehab_v12i1e62986_app1.docx]

| **Introduction** |  | Introduce each other!  Interview structure  Questions |
| --- | --- | --- |
| **Daily life, needs and coping strategies**  **15 minutes** | **Activities and routines** | The type of sports practiced, how often?  A weekly routine before cardiac rehabilitation?  A weekly routine formed during/after cardiac rehab?  How does exercising makes you feel, what do you value in it? |
|  | **Good and bad days/periods** | What constitutes a bad day/period? What are causes of these days and are concerns on these days? |
|  | **Challenges in everyday life** | What are challenges you encounter regarding managing your own health/everyday life? |
|  | **Coping strategies** | How do you deal with these challenges?  What works and what not? |
|  | **Values in life** | What are the things you consider most important in the way you live and work (i.e., your values)?  While striving to keep healthy – what is most important need for you when managing your health *(e.g., keeping a good mindset, having the support of my family, being able to speak to a clinician when I’m worried etc.)*? |
|  | **Exercising and sports** | Why do feel the need to be active?  *Elaborate depending on answer: What does practicing [the sports they do] mean to you? What do you value most about doing [the sport they do]?*  What place does [the sport they do] have among your life priorities *(e.g., next to work, family, or other things you consider important)?*  *Elaborate depending on answer: why is that? What needs does sports fulfill for you?*  Do you practice sports alone or with someone else? Why do you feel the need to do so *(i.e., practice alone or someone else)*?  What role does your social circle *(e.g., friends, family, peers, colleagues)* play in exercising? Is it a passive role or an active role? |
| **Diagnosis, intervention and cardiac rehabilitation**  **25 minutes** | **Changes in behavior and lifestyle** | Please describe one big lifestyle/behavior change you feel you had to make after your myocardial infraction.  *Elaborate depending on answer: is there any other change that you can describe? Why do you feel like this was an important change? In which way did it affect you?*  *(optional) What were the easier/more enjoyable changes in behavior you had to make during cardiac rehabilitation?*  *Elaborate depending on answer: Why do you feel like it was easy for you to change that? In which way did it affect you?*  *What were the harder/less enjoyable things you needed to change in your lifestyle?*  *Elaborate depending on answer: What made it harder to do so?* |
|  | **Program and exercising during the program** | Please describe one time/moment when you thought the program is really helping you in dealing with your myocardial infraction and the lifestyle changes that followed.  *Elaborate depending on answer*: *What aspects made this easy/enjoyable for you? Why?*  Please describe one time when you really had a hard time after you infraction or/and during cardiac rehabilitation.  *Elaborate:* *What aspects made this easy/enjoyable for you? Why?*  Please describe the exercise program of the cardiac rehabilitation in a few words. How did it feel for you? Was it engaging and motivating enough for you?  *Elaborate depending on answer:* ***If it was****, then how did you exercise needs change compared to before your infraction – considering your exercise levels were higher before****? If it was not*** *– what would you have needed for it to be enough for your exercise needs?* |
|  | **Practicing [the sport they do] during cardiac rehabilitation** | Regarding [the sport they usually practice] – how did that change once you started cardiac rehabilitation?  *Elaborate depending on answer:*  *What kind of exercising changes have been prescribed to you during cardiac rehabilitation? How does your exercise routine look now compared to the beginning of the program?*  How did you adapt to those changes – was it difficult to do so?  *Elaborate depending on answer:*  *If it was, then what have been some obstacles mental but also physical that you had to overcome when adapting to these limitations?*  Regarding [the sport they practice] – do you feel like cardiac rehab offered you an opportunity to practice [the sport they practice] or to train for it?  *Elaborate: If it did – how did it do that? If not – then what was missing?*  How was your motivation to practice [the sport they do] during and after cardiac rehab impacted?  If you could adapt cardiac rehab for [the sport they practice] and create your own program – how would it look like?  *Elaborate depending on answer: Why would you make those adaptations?* |
| **BREAK (If needed)** |  |  |
|  | **Information flows during cardiac rehab** | Regarding received information about how you can manage your health (i.e., eating, coping, exercising) after the infraction – what was the most valuable and important information you received?  What were some challenges while obtaining the right information about health behaviors during the program?  *Elaborate depending on answer: What do you feel like you needed in an ideal scenario?*  Regarding information about [the sport they practice] – such as what you allowed to do/what you allowed to do – how was that communicated to you?  *Elaborate depending on answer: Do you feel like you needed more/better information about that at some points? If so – why and in which sense?*  Listening to one’s body vs. adhering to the recommendations – how do you usually approach that? Do you respect the doctor’s recommendations or value your need to exercise more?  *(optional) Do/Did you feel like the information regarding long-term exercising after cardiac rehab was enough? If not, why?* |
|  | **Relationship and communication with clinicians** | The relationship with whom from the clinical staff did you find the most useful and valuable for you?  *Elaborate depending on answer: What did you value in the communication with that clinician?*  Please describe a situation/a series of situations when you really thought the communication with a clinician went well.  *Elaborate depending on answer: Why did you value that interaction?*  Please describe a situation when you thought the communication did not go as you needed/wished for (e.g., some people do not like getting stuck in a line on the phone, or do not feel like their questions are answered in a consultation).  *Elaborate depending on answer: What could have gone better in that situation?* |
|  | **Receiving reassurance** | At which moments during cardiac rehab did you need to receive reassurance from the clinicians?  *Elaborate depending on answer: Why did you feel that?*  Do you feel you received enough reassurance/encouragement from the clinicians during cardiac rehabilitation?  *Elaborate depending on answer: Why did you feel that?* *What did you do in that situation and what would you have preferred to happen otherwise?* |
| **Social support** | **Exercise and social support** | How is your family involved in your exercising behavior? How did your family experience the change in exercise and the diagnosis?  What was the role of the family before cardiac rehabilitation vs. now?  *Elaborate depending on answer: How do you feel about that? Dis you share your struggles with them? Were they involved in the rehab process? Did they influence you in a negative or positive way?* |
|  | **Important persons** | Who is the most important person in this journey? Do you share struggles/frustrations you have about your exercise journey with them?  *Elaborate depending on answer: Why do you think it is important to share/not to share with them?*  What is important to you in relation to the people at home when it comes to your health journey? How can they participate to help you? What works for you and what not? |
| **Technology for managing one’s health/performance**  **15 minutes** | **Types of used technologies** | Do you use any technologies to manage your health/sports performance (e.g., apps, trackers /wearables, blogs, internet)? It could be about tracking but also looking up information or support.  *Elaborate depending on answer: Why do you need to use them? How do they help you?*  What do you like about the technologies you use now? What don’t you like about them?  Did your technology usage change after the myocardial infraction?  *Elaborate depending on answer*: *Why do you think your needs in terms of technology changed?*  ***(If they track their performance)*** *Did you notice any change in the way you use the device after the infraction? What was important to you before and what is important now?* |
|  | **Human-data interaction** | **if they track their data**  What do you value most in your data? What do you like to look at when it comes to performance vs health?  Do you share any of your data with other people?  *Elaborate depending on answer: If so, with whom do you do it and for which purpose? If not, then why don’t you share it?*  Did you ever share your data with your clinician? If yes, in which form (e.g., you show them your phone in consultations, or you generate reports, or send it to them by e-mail)?  *Elaborate depending on answer: If no, why don’t you do it? If yes, why is it important for you that your clinician sees it? How important is clinical validation of the data, especially in cardiac rehabilitation?*  *(optional) What do you do if you see that there are worrying signals in your data?*  *Conflicts between what data shows vs. how their body feels – do you encounter those situations? If so, how do you react?*  **if they don’t track their data**  SCENARIO: If, for example exercising during cardiac rehabilitation was done in the home environment based on whatever sports you would like to do, and the doctor would have to monitor your exercise from a distance through a wearable technology…  Would you use this technology? Would you also find it useful for yourself? Elaborate why.  Would you share any of your data (for example how much exercise you did in a day, your heartrate) with other people?  *Elaborate depending on answer: If so, with whom do you do it and for which purpose? If not, then why don’t you share it?*  SCENARIO: Walter loves to swim 800m once every two days. However, his doctor recommended that he should only limit his swimming to 500m twice a week. Would you share your swimming data with your clinician if you were Walter?  What if you got a warning that your heart is beating abnormally high from your smartwatch – would you share that with your clinician if they could react at some point?  *Elaborate depending on answer: ask for more explanation of why and how* |
|  | **Cardiac telerehabilitation technologies in cardiac rehabilitation** | SCENARIO: If, for example exercising during cardiac rehabilitation was done in the home environment based on whatever sports you would like to do, and the doctor would have to monitor your exercise from a distance through a wearable technology…  **if they track their data -** In the case of cardiac rehabilitation at home, should the technology used for tracking exercise be devices you already use or devices provided by the hospital?  *Elaborate depending on answer: ask for more explanation of why and how*  How would it make you feel If clinicians would have your exercise data and could react on it and give you advice?  What would be some obstacles for you to use such technology in cardiac rehabilitation at home – for example, some people like the freedom of doing their sports at home and being supervised from a distance, other people do not trust these technologies or think they involve too much effort – which one is you? |
|  | **End of interview** | What is the most important to consider when  designing a system that can allow master athletes to have more personalized cardiac rehabilitation in the home?  Anything missing or that has not been  mentioned?  Other ideas or questions? |
