# Supplementary material for: Design Requirements for Cardiac Telerehabilitation Technologies Supporting Athlete Values: Qualitative Interview Study
Source: JMIR Rehabil Assist Technol. 2025 Apr 17;12:e62986. doi: 10.2196/62986 (PMC12046260; doi:10.2196/62986)
Supplement: Multimedia Appendix 2 [file rehab_v12i1e62986_app2.pdf]

Consultations, coaching and guidance

Consultation with clinicians

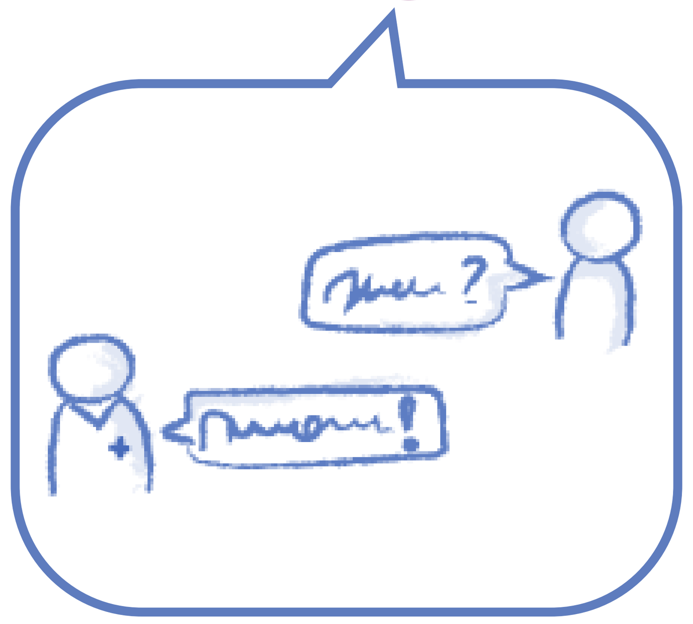

Having periodic checks/ consultations (e.g. with a clinician, periodic surveys or with a virtual agent)

Being able to request on-demand consultations whenever I need to (e.g. with a clinician, through a survey or with a virtual agent)

Having remote consultations (e.g., by phone, through texting or video calling)

Having in-person consultations with a clinician

Other

Supervised training

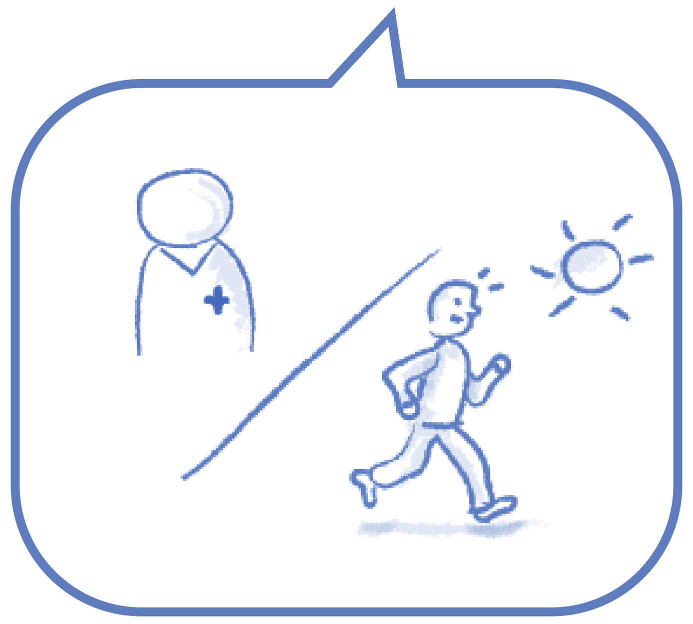

Training independently at home (doing my own sports), with periodic checks on my workouts through remote supervision

Training in a group (e.g., through video conferencing or face to face), doing standardized cardiac rehabilitation exercises, with live supervision

Being able to provide feedback to clinicians about the training sessions- e.g., how I felt during the training and how it can be improved

Being able to include my family/friends to participate in trainings with me

Other

Receiving feedback

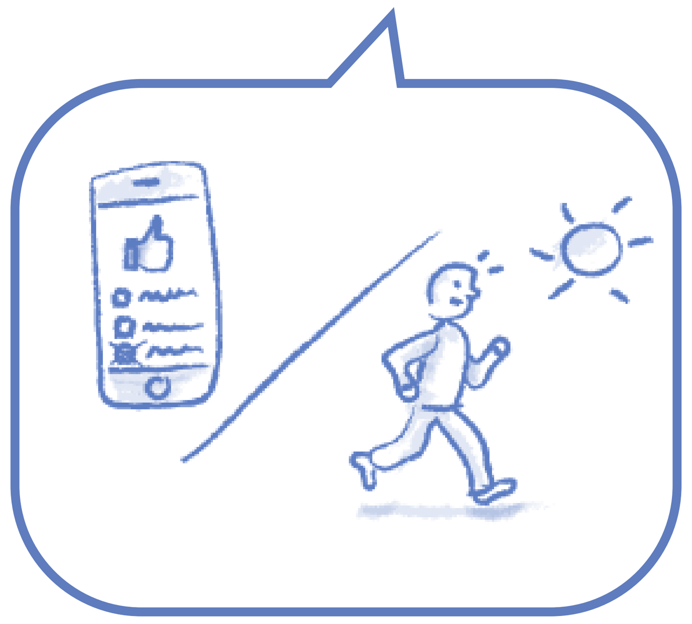

Receiving personalized clinical recommendations and planned activities (e.g., training schedules, limitations for how much I can do)

Receiving feedback on my performance and health data during my workouts

Receiving support from clinicians regarding my emotions and worries

Receiving feedback from clinicians when there is a red flag in my data (e.g., heart rate too high for too long)

Receiving periodical feedback and recommendations from a virtual assistant (i.e., an automated system) on my current behaviors and health data

Other

Sharing data with clinicians

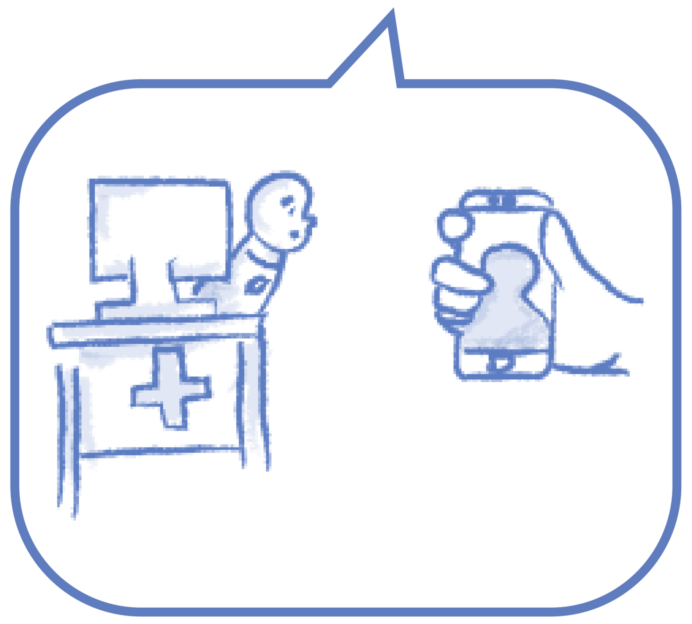

The system automatically sends my data to my clinicians (e.g., in real time, or periodically)

I am able to manually send my data to clinicians whenever I want to

The system notifies clinicians when there are red flags in my data (e.g., heart rate is too high for a long time, or performance is above limitations)

Being able to show representations of my data during consultations to my clinician (e.g., to discuss about it or ask questions)

I am able to choose myself which data my clinician is able to see (e.g., only showing activity and sleep, hiding stress)

Clinicians can receive and see all the data collected by the system

Other

Monitoring behaviors for supervision and oneself

Entering data

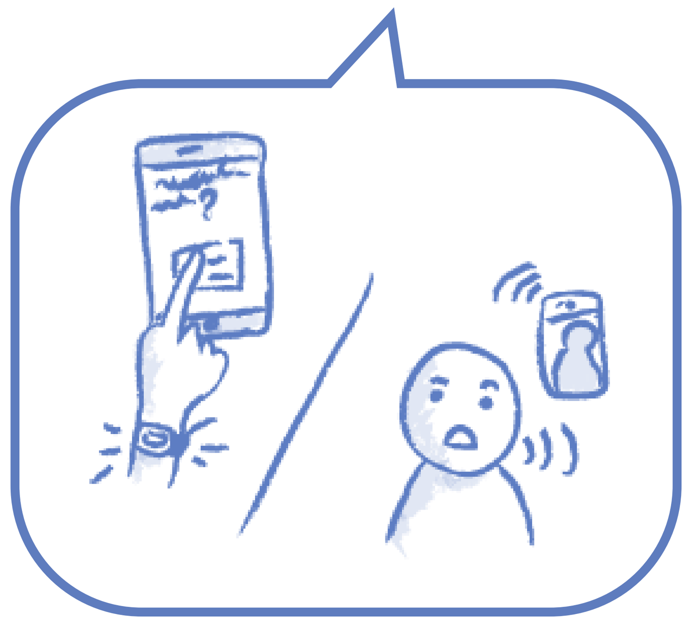

Using technology to track my activities and behaviors- e.g., mobile phone, a wrist sensor, chest strap or health patch

Reporting on subjective experiences through diaries or surveys- e.g., emotions, worries, side effects, stress, social interactions etc.

Having periodical reminders if data has not been entered for a while

Other

Setting goals

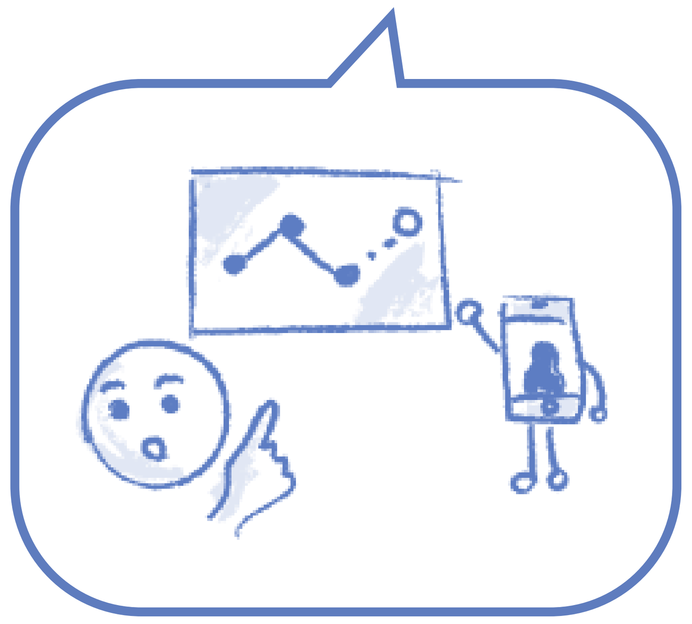

Setting short and long-term goals for myself- e.g., how much sports I do per week, at which intensity

Receiving personalized training goals from my clinician (e.g., goals based on my previous athletic performance)

Collaboratively adapting my goals together with my clinician based on my personal situation

Having rewards like points or trophies when achieving certain goals or targets

Having periodical reminders about targets or habits to work on

Having periodical motivational messages on ‘bad days’ or when almost reaching a goal

Other

Interpreting data

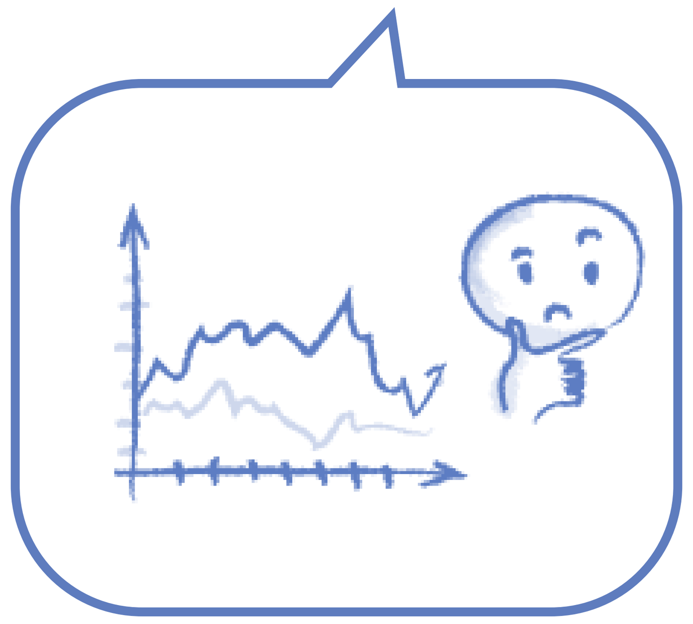

Seeing performance and health data in a graphic form- e.g., graphs, animations, icons etc.

Seeing comparisons between performed workouts versus my clinical recommendations (e.g., suggested max heart rate next to your actual heart rate)

Receiving notifications when there are red flags in my data (eg., heart rate is too high for a long time, or performance is above limitations)

Other

Co-experience (social aspect)

Sharing data with others

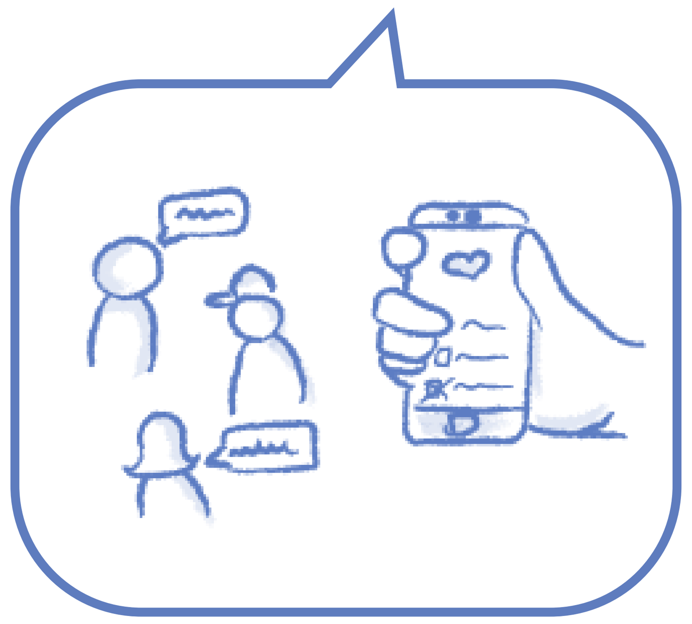

Sharing my data with my family and/or friends (e.g., for discussions, sharing concerns, making decisions together, planning activities)

Sharing red flags in my data (e.g., heart rate is too high for a long time, or performance is above limitations) with my family or friends

Other

Connecting with others

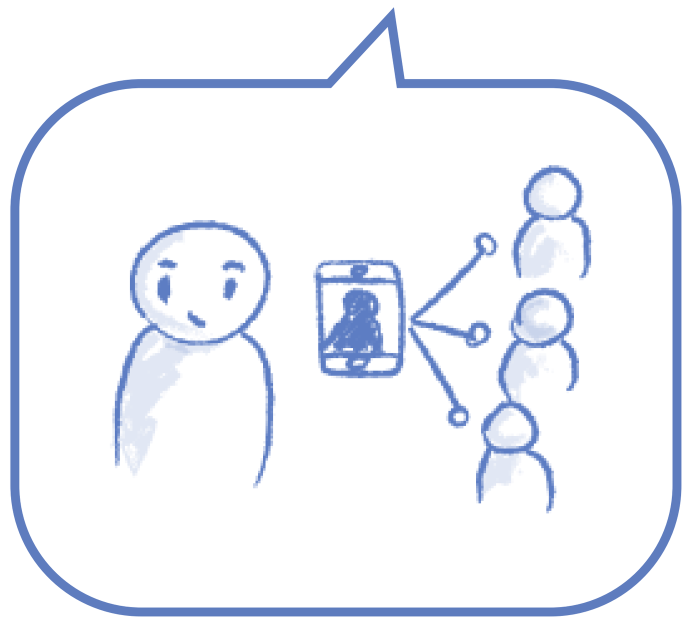

Connecting and communicating with other patients through an online community (e.g., for communication, sharing experiences, cooperation)

Receiving peer-based support from other athletes with cardiac problems- e.g., on my training or my trajectory

Having means or channels that allow me to train with other peers (e.g., other athletes with cardiac problems)

Other

Education and assistance

Onboarding and support

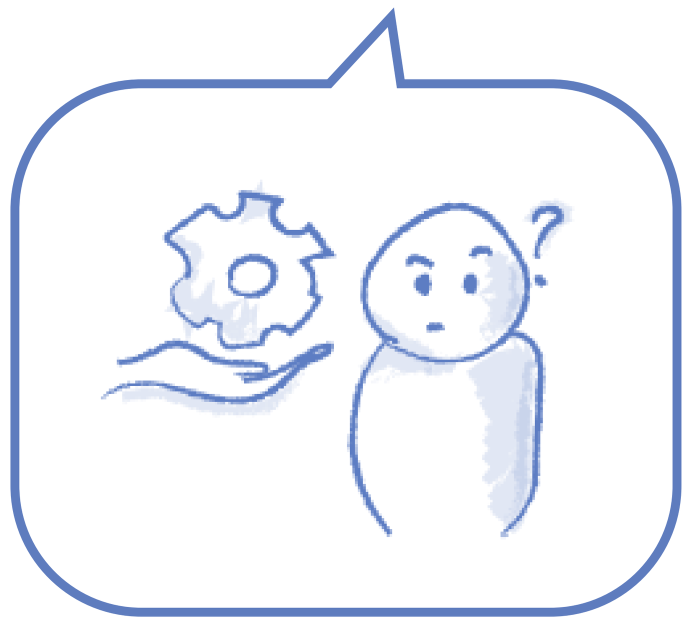

- Using the technology I already own (e.g., mobile phone, wearable- watch, breast belt etc.)
- Having the hospital provide me with the technology I need (e.g., a wearable sensor and a tablet/mobile phone)
- Instructions about why it is important to monitor myself and how it can benefit my health
- Technical assistance and instructions on how to use the technology, share the data etc.
- Being able to personalize the technology (e.g., making my own avatar or setting reminders and feedback based on my own preferences)
- Other

Receiving education and information

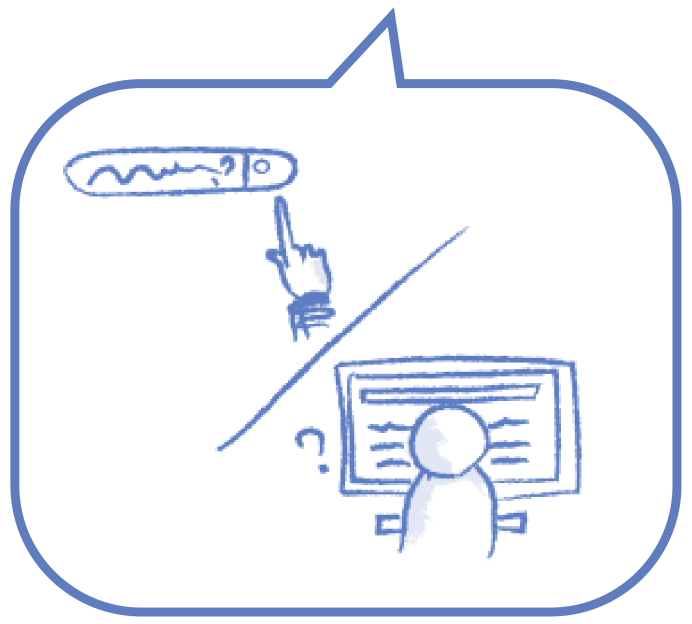

- Having access to educational materials in digital form (e.g., videos and tutorials, information webpages)
- Being able to take part in educational group sessions moderated by a clinician (e.g., online or face to face)
- Asking for information from a virtual assistant (e.g., automated chatbot)
- Being able to search for clinically validated information anytime I need to (e.g., about my condition, medication, fears)
- Other
