# Supplementary material for: Design Requirements for Cardiac Telerehabilitation Technologies Supporting Athlete Values: Qualitative Interview Study
Source: JMIR Rehabil Assist Technol. 2025 Apr 17;12:e62986. doi: 10.2196/62986 (PMC12046260; doi:10.2196/62986)
Supplement: Multimedia Appendix 3 [file rehab_v12i1e62986_app3.docx]

Values and needs of Athletes within CR

Codes

| Value and underlying needs | Description | Example quotes (illustrative) |
| --- | --- | --- |
| **[Value]** Health and performance quantification | | |
| **[need]** measure progress and performance | Athletes use data to measure performance in their everyday life and exercising – wearables, mobile and web applications; they look at HR, distance, speed, and time. They also like to have indications that their body is making progress during recovery – exercise measurements in the training program and verbal feedback from clinicians. They would like a more structured way to have an overview of their progress with both data from wearables and program measurements. Some athletes use technology for exercise scheme suggestions (e.g., ChatGPT, Strava, Garmin). | *“I would immediately make a bridge to technology [i.e., for the rehabilitation of athletes]. Almost all of them have smartwatches or something, so they are very driven by numbers, so they prefer to train at a certain frequency.” (HCP5)*  *“I gained some. That's what she said, you've improved your wattage quite a bit. We are very pleased with what you have achieved so far. So that was enough to take home.” (P1)* |
| **[need]** routine measurements to check in with one's body | Athletes perform routine measurements with blood pressure monitors at home. They also appreciate the bike tests, as well as other measurements done by clinicians - they trust the medical apparatus; these measurements give them an indication of where they stand and make them feel looked after | *“It is important to measure, where are you now. […] Blood pressure, heart rate. I think that's important, it reassures you when they [i.e., clinicians] measure, so that's going well. I can exert myself again, that measurement is good. So that gives confidence. Confidence in your own body gives you another chance to take the next step.” (P5)* |
| **[need]** self-monitoring for limitations in exercising | athletes learn how to set limits for themselves - and sometimes they use measurements from wearables and computers to position themselves and know when to stop and take rest | *“I used to wear a band when cycling to keep an eye on my heart rate. It's just, that keeps track of what I want. 155 and done, no more.” (P1)*  *“[…] to measure while running. How far can you go, where is the limit. I'd like to know that actually. And then start very slowly at a slow pace.” (P6)* |
| **[Value]** Coming to terms with one’s condition | | |
| **[need]** accepting the diagnosis | Athletes need to come to peace with their diagnosis and accept the lack of control over their condition that is mostly associated with unhealthy lifestyles (that does not represent them). Most athletes mention a family history of heart problems - they realize it is not anything they did 'wrong' even though they try to find an explanation. As they have been active almost all their life - sports is something they identify with; the heart condition makes them weak and fatigued; they need to accept that their body is less able. | *“I have also seen a man who was really in his late seventies somewhere and he could hardly accept that he could no longer do certain things, while most men his age have already passed away.” (HCP4)*  *“That doctor who was at my bedside knew that I was a runner. We've talked about that before. He himself said ‘how is it possible?’. […] How could I have ended up here? […] The average there [i.e., patients in the hospital] didn't look very healthy after all. So then you lie there and then you think how is that possible. […] you think it's very unfair. Also because you live a healthy life, you exercise a lot, etc.” (P8)*  *“I think 8 hours. [i.e., of sport perf week]. […] There was nothing wrong. So that's why I ask myself ‘did I do something wrong with sports? Did something go wrong? How come I got this while I'm a sports fanatic?’” (P13)* |
| **[need]** adapting to new capabilities and setting boundaries for oneself | Athletes' goals are not only going back to performance capabilities, but also enjoying the dynamics of ordinary life as it was before the cardiac intervention. Athletes need to learn how to stop themselves from pushing themselves too hard, slow down, and set boundaries for activity performance. Athletes also experience extreme fatigue, especially in the beginning of CR - something they are not used to, and they find coping mechanisms to deal with it; they go from 100 to 0 after the heart event and have to deal with being incapacitated for the first time without guidance after the heart event - this takes a toll on their confidence and mental health. | *“you [i.e., the athlete] miss the challenge because they are already so passionate about sports, so it is always good to give advice about what they can or cannot do in terms of physical exertion. And advise on it.” (HCP5)*  *“That's [i.e., the gym session] an hour, an hour and a half each time. Then it's okay too. But I have to pay attention.*  *[Interviewer: What do you have to pay attention to?]*  *That I don't end up in a dip, because I've had that once. That I was going too fast. That the recovery takes longer to be able to start exercising again, let me put it this way. (P7)”* |
| **[need]** dealing with family anxiety | Family is generally more anxious than the patient; they try to discourage them from doing too much physical effort, and they are worried when patients sport alone in remote areas - patients and family sometimes negotiate and find safe ways to exercise (e.g., using app to share one's location with family member) | *“they [i.e., family] are sometimes marked or they are more anxious than the athletes. Because of course they themselves [i.e., athletes] feel how it [i.e., the symptoms] feels. And the environment [i.e., their close ones] just has to wait and trust that.” (HCP7)*  *“my wife would say ‘take it easy, be careful!’. So she's a bit more reserved about that. By the way, my daughter too, with her new heart. She says ‘dad, you must take it easy’. That was always said to me. Then I say ‘I will’. And I have done that, I think, subconsciously I have been working on it anyway.” (P5)* |
| **[need]** ability to recognize symptoms | Athletes are mostly asymptomatic before their diagnosis and need well-tuned and well-informed mechanisms of recognizing symptoms by listening to their bodies and coupling the ‘feeling’ with concrete data and indicators. | *“It took way too long for the helicopter to come. Not because that helicopter didn't come quickly, but because I reacted too late. I was on a bike; I was on a slope and I wanted to keep up with some people. But I just didn't expect to have anything going on with my heart.” (P2)* |
| **[Value]** Independence and confidence in one’s body | | |
| **[need]** ability to exercise at home | Athletes have a higher standard for 'being active' compared to sedentary patients, this standard includes being able to exercise the same way, or similar way to how they exercised before hospitalization - they need to learn to exercise in uncontrolled environments again. This also includes being confident in the signals their body is sending and dealing with exercise-related anxiety. | *“Sometimes there is also some fear. That they think, I used to be able to go up to a heart rate of 200, do I still dare to do that now, is that still allowed.” (HCP8)*  *“I said ‘if it [i.e., the rehabilitation] goes well I don't really want to come here twice a week anymore. Then I want to go to the gym and can you advise me what to do with this.’ Then they said ‘first go to the gym and exercise under supervision’. And I did. The gym guided me, that went very well. So then I didn't have to go back [i.e., to the hospital exercise program].” (P13)* |
| **[need]** control over one’s life and health | Always staying 'busy', perceiving inactivity as negative and associating activity with feeling in control of one's life and health. | *“I have to keep doing activities. A bad day means that too little was actually done. Don't get lazy.” (P2)* |
| **[need]** confidence in oneself and one's goals | Athletes know what they want to get out of rehabilitation – control over their life and fitness. For some athletes, they fall back on interpreting data after checking in with their body - they are sure of what their body can do and reading its signals, so they check in with their body first. | *“We don't see that many athletes, because you often see them for a very short time, because they are independent very quickly.” (HCP7)*  *“I didn't have any anxiety or anything like that, so I didn't need reassurance that I could do that. […] There was also not a lot of motivation required, so they didn't have to tell me how you can you do that.” (P9)*  *“I don't care much about that kind of data. I listen carefully to my body. And when that body says, that heart beats too heavy. Then I know I have to go back and start recovering. And that goes pretty well too. So I don't think that data from my watch is very important.” (P4)* |
| **[need]** self-sufficiency to help others | Many athletes feel the need to remain healthy to help and be next to people around them - family, volunteering organizations etc. | *“I mainly look at myself. I want to be good to myself. I have a slogan, if I am good to myself, I can do many things for other people. So that's kind of my basic attitude. I would like to be among people, but I first look at myself, what can I do well, I also want to do well.” (P5)* |
| **[need]** vitality and athleticism despite age | Older athletes are proud to be fit despite advanced age and want to remain the same and defy the limitations imposed by their condition. | *“There [i.e., at the rehab program] were people who were not that active. […] There was a woman who said ‘I wish I could to that.’ [i.e., what the athlete was doing].*  *[Interviewer: How did you find that?]*  *I was very proud. And also with cycling. […] It's good to get a compliment.” (P13)* |
| **[Value]** A dynamic lifestyle | | |
| **[need]** social bonding through sports | Some athletes need the social aspect and bonding of sports and competitiveness. Some athletes, on the other hand, prefer to sport alone because they can push themselves in their own rhythm - they are in competition with one's capabilities. For some, social sporting also provides a way to be safe. | *“We didn't have to say anything to each other during sports, but especially the idea that you are together. And even if something happens, there is always someone there.” (P6)*  *“I always find it a fun challenge. I must say the challenge with table tennis is a bit bigger, because then of course you want to beat other people.” (P2)* |
| **[need]** a way of remaining healthy | Sport is associated with being healthy - athletes exercise to stay vital and independent. Most athletes place sport very high among life priorities - mostly because they associate it with health and wellbeing. | *“Just to keep fit, that's all I want. I think I weighed 130 kilos, but I wanted to weigh 95, back then when I didn't exercise intensively.” (P7)* |
| **[need]** sports as part of one's identity | Many athletes have been sporting for a really long time, sporting becoming more than just a hobby, a part of who they are as a person, something they identify with. | *“When I was young, my father told me or said to our family ‘there are three important things: nutrition, sports, and education.’ When I was five years old, my first sports was taekwondo, until 11, and I'm going to gymnastics, [name of sports club]. They saw that I was talented. So, I sported at a high level on [name of sports organization], till my 26th. After that, I have a lot of swimming and [unintelligible]. After that, I did a lot of fitness, cardio and hit.” (P11)* |
| **[need]** a psychological coping mechanism in life | Some athletes use sport as a way of coping with everyday struggles, it is therapeutic, a way of meditation. | *“I did it [i.e., sports] from when I was raised with sports. But also, it's my way of getting rest in my head. Otherwise, it [i.e., the mind] goes on the whole day, even in the night. The way to stop it - catch rest in my head is by doing sports.” (P10)* |
| **[Value]** Care with ’a personal touch’. | | |
| **[need]** human interaction | Although athletes are more independent, they still appreciate personal approaches to healthcare - having face to face conversations and a personal approach. | *“I think that the therapist should get into contact with people more by asking more questions. You are put on a bike, and then they come with the heart rate monitor. They're all busy, I know that.*  *[Interviewer: But is personal attention important to you? And whether that is in the hospital or in your area, it does not matter?]*  *Yes Yes. They don't have to talk for an hour, but a short go-around would be nice.” (P6)* |
| **[need]** emotional support and sharing (clinicians) | Some athletes deal with anxiety after the cardiac intervention - their body failed them and they struggle to accept their condition; some athletes appreciate communication with clinicians that address their emotional struggles. | *“there are also patients who are a bit anxious, especially if it is an event [e.g., heart attack] that happened during sports, that makes it a bit more challenging. And I think that's actually a group where I think more often, it would be nice if we had sat opposite each other for a while.” (HCP3)*  *“you can pull out the emotional aspect in the person. That is often locked. And when that is locked, it can do a lot more to your heart - problems I think. I mean if you can't figure it out on an emotional level, you're going to have a problem with that.” (P1)* |
| **[need]** reassurance, encouragement and feedback (from clinicians) | Some athletes need reassurance through progress reports, feedback and encouragement that there is nothing to worry about. | *“We had an interim conversation. After 7 lessons approximately. We've had that and then it was pretty good. They said ‘you're doing really well in the group!’[…] So nice to hear that too.” (P4)* |
| **[Value]** A goal and performance-oriented approach | | |
| **[need]** challenging oneself | Athletes like to push their limits slowly and steadily in order to maximize one's capabilities and regain confidence in what their body can do. They want to improve their physical condition no matter what, and they generally accept any intervention that would help them do so. They are already very motivated; they do not need any push to exercise; however, they might need that in the beginning, right after the intervention when their body is not able to do much. | *“I don't know how far they are always able to adjust it themselves, because their willpower is very high and then they may want to achieve their plan at all costs.” (P5)*  *“anything that would get my condition back, I would have done. If it were, I don't know, if it was something completely different I would have done that. […] for me it was definitely the first steps to be able to do something, so it was really important. So the motivation was very high. I never missed a single training. I always was there. So yeah, I think it was very good because I saw that. And in the beginning I was walking on the treadmill and I did more intervals and intervals became longer and that is incredibly motivating. ”(P9)* |
| **[need]** exercises adapted to one's high fitness | CR exercise is not intense enough for athletes - they need exercises that push and challenge them. For some participants, the exercise program was so underwhelming, that they did not find it useful for their own recovery; they did it to make sure they do not 'skip' anything important for their health and for the social aspect. | *“I think it's still too much uniformity. So you go to physio and you train like the rest of the group. You will get quite a bit different power[…] But I think that the fit, active man or woman may need something different than the over eighty. So maybe a little more challenged, just a little more tailored.” (HCP9)*  *“I understand that there are few people like me who really want to exercise. So they can't wait for a group of people like me to get together. So it is very understandable that you join those [i.e., the more sedentary] people. I've played games where I thought, does all this make sense for me?[…]For me that is very little. But on that bike, nice cycling, and also on that treadmill, that was nice. The physiotherapists who were there, allowed me to go pretty fast there. But actual it's not motivating to exercise I can tell. Before the interview I was also thinking, how could you do it differently? On the one hand, I want to start as early as possible. And preferably with six people like me. But yeah, you can't do that.” (P8)* |
| **[need]** returning to previous athletic capabilities and goals | Once they start recovering, athletes are eager to go back to their previous performance goal. They prefer setting their own goals. This includes being able to sport in their own home environment to achieve similar performance. | *“The real athletes just want to return to the level they used to be” (HCP1)*  *“What I often notice is that they are also very motivated […] to start things up again quickly in the home situation and then they actually ask us ‘I want to start again, knowing what can I handle, what can I do and how does my body feel during exercise.’” (HCP6)*  *“I cycled before covid and last year for charities in France. [names of the mountains]. I want to do that again. I like that.” (P7)* |
| **[need]** seeing physical progress during CR | Athletes like to have indications that their body is making progress - numbers, verbal communications from clinicians, and a history of performance indicators to compare it to themselves. Athletes generally only relate their performance to their own past performances, however, exercising with less fit people in CR makes them feel proud about their progress. | *“At one point, when they showed the board, they said, what grade do you give. Reasonable. Not heavy or strong or anything like that. Oh, then next time you can cycle 5510, that's good. And, that went fine. And then I think I go it. We keep going, improving the condition.” (P1)* |
| **[Value]** Clinical validation on information and data | | |
| **[need]** checking sensor data with clinicians | For some athletes, data from wearables is not enough - athletes want clinicians to look at the data and find validity in it based on their specific condition and clinical tests. However, some athletes feel like clinicians do not give enough attention to this aspect. | *“I now also have that blood pressure, and then it was above 100, the overpressure. So I do share that. I'll keep an eye on it a bit. I don't think they [i.e., clinicians] care that much." (P2)*  *“they [i.e., athletes] want to share the information anyway. They tell us about it. It is better to be concrete, have it on paper, objectively and in an insightful way.” (HCP3)* |
| **[need]** discussing implications of data with clinicians | Athletes want to share data reports with their clinicians and get insightful feedback based on it. | *“[Interviewer: Do you see advantages or disadvantages in sharing data with clinicians?]*  *Only advantages. That there is a little more guidance. Because I have that guideline of 130. Because I would like to take that step, so that I can go a little further. I sometimes think about joining an athletic club again.” (P15)* |
| **[need]** receiving clinically-validated information | Some athletes have the tendency to not trust random sources of information, but only the information received from the hospital or provided by clinicians. They values the insights from the hospital educational materials. | *“But if it [i.e., information from the hospital] is aimed at athletes and certain heart problems and rehabilitation, then it might be useful. [..] In the beginning, when the rehabilitation had not yet started, I already googled what about sports. But then you get a bunch of different websites saying different things.” (P3)* |
| **[Value]** Concise, actionable guidelines | | |
| **[need]** transparent indications of symptoms | The athletic population is generally asymptomatic, they also show symptoms during performance, and they tend to confuse symptoms with performance pain - they are confused about recognizing symptoms and are afraid of getting another heart event - they want to know exactly what to look for and need high transparency when it comes to predictions of other heart events. | *“I also had an important question ‘how big is the chance that I will get this again? Is this bigger now or not?’ Those are important questions to me. He had a computer program for that, [name of the program]? That just didn't make sense to me at all.” (P2)* |
| **[need]** clear, quantifiable exercise limitations | Athletes need to understand how far they can push themselves during exercise, they want clear indications of what the max HR is for them, how safe it is to push limits, what “if it feels right to you" means exactly. Sometimes, they need progress reports to relate back to the exercise limitations. Athletes place their health above performance, so they put clinical recommendations above anything else; however, they are very sure of themselves and know their body very well, so they combine the two: intuition and recommendations. | *“the most important thing is ‘what can I do, what can't I do’. Which is talked about a lot of the time. But also that aspect I just mentioned. Gaining confidence in the body again. So what can I do. And to rebuild it. And I think that we can certainly play an important role in this with spiro ergometry, which also offers some reassurance in this.” (HCP3)*  *“Because now it was, me guessing what I could do. And then asking you know ‘is it OK if I run?’ and then the answer would always be ‘yeah. If you feel like you can do that, you should do that’. It's the same as the cardiologist always says. You know, if I ask him ‘OK, can I do that again?’ Yeah, that's not very precise because it is a heart condition, right? So you don't want to overdo it.” (P7)* |
| **[need]** tailored exercise plans | Athletes feel the need for adjusting recommendations and exercise sessions to their intensity in order to make it feel like exercising is actually helping them and challenging them – e.g., adjusted weights, workouts based on their medication and personal situation; athletes who do not receive this from their clinicians find work-arounds: ask their PT or their network (e.g., son is sport scientist so they make personalized exercise plans for them). Some athletes feel like they are not treated with serosity because they are active and fitter than the general population, so they are ignored by clinicians and told they 'will be fine' - however, they still feel the need for guidance and reassurance, as well as attention. Some athletes need at least some indication of how to continue exercising after CR; however, the need for that is less as time passes, opposed to the generic population - once they learn their new capabilities, they need less and less guidance | *“People who are usually very fit physically. And if you were to blindly send someone like that here for a normal heart rehabilitation process, I don't think it always benefits. Because they actually need a completely different process. And sometimes I do send those people for a short rehabilitation, but I try to set it up a little differently. Looking together with the sports doctor to see how we can best guide such a person.” (HCP10)*  *“Maybe a plan, saying you know, so that's your goal. So what we will do is, we will offer you this. We have a joint thing with a group. And in addition maybe do not do the same I did with that training center. You know, here is a program that will start. But in three additional months, we'll get you back to the condition I was closer to, what you started. Yeah, that would have been great.” (P9)*  *“But when I was outside and I got hyperventilation, I was afraid. I called my sister-in-law, of course, she's also a patient, and she got me through it. After one week at home, I'm going to the gym only to talk. I asked a friend of mine, he's a personal trainer ‘Can you help me, please?’ He said ‘okay, no problem’. We started by walking for only 10 minutes - then I said, ‘Okay, it's okay’. Yeah, I build it up every week. Now I sport six days a week, so I ask my cardiologist ‘is that okay?’ He said, ‘Yeah, perfect.’” (P11)* |
| **[Value]** In and outside hospital oversight | | |
| **[need]** earlier supervision and guidance | Athletes express that they need the most care after the cardiac event/diagnosis - that is when they are the most incapacitated and have the most questions; most participants wished they started the program or being supervised immediately after hospitalization - many found workarounds within their own network and resources to re-start moving in the gap before CR. | *“Communicate faster with each other. It happened to me that I was forgotten. Well, that shouldn't happen. […]I mean, such a heart attack has been on [date]. Then it shouldn't take too long, not 3 months before you start such a rehabilitation. That should be much faster. Because in those 3 months a lot can happen to such a person.” (P1)*  *“Ridiculous, I had three or two intakes [before starting the program]. But now you're in the program and now you get another intake. It's ridiculous. I think you tell the same story. I could have started with the physio today. And to tell the same story, […] And first intake was also from a physio. The second intake was also from the physio. […] You lost a month again!!” (P12)* |
| **[need]** feel looked after by a healthcare professional/ ‘be part of something’ | Some athletes are insecure after hospitalization and participate in the program because they need to know that they are looked after by a health professional; they feel safer when they know they are being supervised, especially in the first 6 months after hospitalization. Although most athletes don't perceive the exercise program of CR as valuable, they still participate because they feel the need to do something for their own health and take advantage of all the available opportunities - they feel more in control of their health journey. | *“They really need it, I have the idea, they like to train under supervision.” (HCP7)*  *“I ended up doing it because maybe I was afraid of being isolated.” (P2)*  *“they kept a very close eye on everything* […] *there is that nurse or that physiotherapist that stands next to you.” (P4)* |
| **[need]** feeling reassured and calm through supervision | Athletes prefer supervision as a way to reassure them periodically that they are on the right path and calm them, provide safety, especially int the beginning of the program. | *“Experience at least a few times under supervision what is possible. And if they realize that and feel what's good, and they like that, often they do the first few training sessions under supervision. Just on the treadmill, just running, just cycling, just experiencing those peaks. And that they can then translate that into practice themselves. But they really like that first piece of guidance.” (HCP7)*  *“Rebuilding trust is often an important component in a recovery. Maybe rehabilitate under supervision in the clinic the first few times and then just remotely. Or perhaps even closer to home, if it can be arranged in such a way that a patient can simply train at the physio around the corner, where they may already be familiar.” (HCP5)*  *“that cycling under supervision, I also thought that ... [unintelligible]. I would start in the hospital, that would give me certainty that I am doing well. Then that could be phased out, so that at a certain point you only do it at home.” (P15)* |
| **[Value]** Trustworthy, readily available support | | |
| **[need]** punctual follow-ups and feedback | Athletes appreciate clinicians who address their problems in short time, swiftly. They appreciate strategic advice and actionable feedback - unconcluded problems make them feel like they have not been taken care of. Some athletes did not recognize or ignored symptoms before the diagnosis or heart event; therefore, they would immediately rely on clinical help if there were worrying signals in their data for a longer period. | *“I had some issues sleeping. And that didn't came from the mind, but from the medicine. But they really took care that, okay, we found some action points, and that was also for the restart of work. And then there were some action points and they really made sure that all action points are taken over, by work or by the other things before they closed the case. So I had several times contact by phone with my psychologist – ‘Here we make sure that all issues are all finally addressed and will then be solved in a proper way. If everything is checked then it really closes.’” (P3)* |
| **[need]**collaboration when making health-related decisionspersonal | Athletes prefer bi-directional care rather than one-directional care. Athletes appreciate when goals are adapted together with clinicians, and they also have control over the recommendations. | *“the people who worked there [i.e., the rehabilitation program], the physiotherapists. […] They're really going to help you and see what program fits with you. […] They asked me what I want. That was nice.” (P10)* |
| **[need]** streamline communication | Athletes need easy, fast communication channels that do not close the communication before their questions are answered and do not take too long to answer simple questions; they prefer digital means for short questions as long as they are reliable | *“I think that the communication with the cardiologist is a bit slow - the E-consult. Every time you send a message and then first somebody else has to check it. […] I put it through and you have to wait 2-3 days and then you'll get the response and then he says ‘okay. Look, do this and come back to me in a week’. And then they only keep the e-consult open for six days and then you want to write something back. And then the e-consult is closed again and then you have to start a new consult” (P3)* |
| **[need]** to have complete trust in clinicians | Athletes are very sure of themselves, however, when it comes to clinical matters of the heart, they completely rely on clinicians - they leave the decision-making to them, and when the clinicians do not give much regard to them, they lose trust and are disappointed. | *“In this I find my doctor's opinion leading. […]I think he just has knowledge in that field. He controls that. If there is something, he will tell me. Yes normal conversations.” (P2)* |
| **[Value]** Emotional support and sharing | | |
| **[need]** being around people with the same condition | Most athletes participate in the program for the social and bonding aspect, for not feeling alone; even participating in the focus groups gave them an opportunity to share struggles and bond with other participants which they found enjoyable and useful. | *“if they have questions, then often when they are on the bike or walking on the treadmill, then I also just start a conversation about how are things going and then everyone will often join in. And at a certain point the conversation just runs between them and then you also hear something and then you also get feedback from each other. And I think that is really the intention when they are training together, especially in the beginning.” (HCP2)* |
| **[need]** opening up and sharing experiences with family and close ones | Although family does not play an active role in exercising and recovery, most athletes share their struggles with their family; some seek this support from the program clinicians. | *“I think very important [i.e., the wife’s role in rehabilitation]. She knows exactly what happened to me. She knows the status of my heart and body, and how it has recovered. That bike test was also very good. She said, your heart could handle it very well, that was already after three months.” (P14)* |
| **[Value]** Reliable information systems | | |
| **[need]** accurate sensors and data | Some athletes struggle with inaccuracies from wearable sensors and do not trust data from non-clinical devices. They do trust wearables that are accredited as medically accurate. | *“[about a fitness wearable] I tried it in the beginning. I have to use it also because it can also measure your blood pressure and ECG and all the things that are interesting during sports. But I don't think it's good enough to get good data from that.” (P3)* |
| **[need]** easy-to-use, understandable systems | Athletes need user-friendly, reliable systems that are enablers not obstacles to managing one's health. | *“I think that watch, quite a lot of people now have a smartwatch. Especially those athletes. And that they can use their own smartwatch. I think that is really an added value. Not that they get another one that doesn't know how it works.” (HCP2)*  *“I know I'm getting a little older myself. I notice, 20 years ago I had no problems with computers. Now the technology has stagnated in my eyes. With AI coming soon, I don't know if I am able to do that.” (P4)* |
| **[need]** robust and simple infrastructure | Exercising at home is preferred if guidelines on using remote systems are clear and transmitted through reliable structures that are simple to use. | *“that platform [i.e., CTR system used during Covid], we had really big problems then, just practical problems, but I think they had to do with data security. I think it had to do with the fact that each device had to upload and download the information for the connection, so to speak. But that made it almost impossible for us to be in a group with a number of patients. We always had problems with outages, with connections, people who couldn't get in. And I really think towards the future, if you really want something like this to work, then those preconditions just have to be really good.” (HCP4)* |
| **[need]** technical support | Athletes need technical support on using technologies for their own health. | *“explaining why we do it, that's where it starts. This encourages use. Explanation and communication is very important . And once it doesn't work and they get frustrated, you lose them.” (HCP7)* |
