# Supplementary material for: Design Requirements for Cardiac Telerehabilitation Technologies Supporting Athlete Values: Qualitative Interview Study
Source: JMIR Rehabil Assist Technol. 2025 Apr 17;12:e62986. doi: 10.2196/62986 (PMC12046260; doi:10.2196/62986)
Supplement: Multimedia Appendix 4 [file rehab_v12i1e62986_app4.docx]

Identified athlete values with paired needs and example quotes from the interview phase

| **Index** | **Value (theme)** | **Needs (sub-themes)** | **Example Quote** |
| --- | --- | --- | --- |
| *Body-centric* | | | |
| V_1_ | A dynamic lifestyle | - social bonding through sports - a way of remaining healthy - sports as part of one's identity - a psychological coping mechanism | *“I was raised with sports. It's my way of getting rest in my head.”* (P10) |
| V_2_ | Independence and confidence in one's body | - ability to exercise at home - control over one’s life and health - confidence in oneself and one’s goals - self-sufficiency to help others - vitality and athleticism despite age | *“I didn't have any anxiety or anything like that, so I didn't need reassurance that I could do that [CR]. […]  There was also not a lot of motivation required.”* (P9) |
| V_3_ | Coming to terms with one's condition | - acceptance of the diagnosis - adapting to new capabilities and setting boundaries for oneself - dealing with family anxiety - ability to recognize symptoms | *“8 hours. [i.e., of sport per week]. […] There was nothing wrong. So that's why I ask myself ‘did I do something wrong with sports? [...] How come I got this while I'm a sports fanatic?’”* (P13) |
| *Care-centric* | | | |
| V_4_ | A goal and performance-oriented approach | - challenging oneself - exercises adapted to one's high fitness - returning to previous athletic capabilities and goals - seeing physical progress during CR | *“Anything that would get my condition back, I would have done. […] So the motivation was very high. I never missed a single training. [...] I did more intervals and intervals became longer and that is incredibly motivating.”* (P9) |
| V_5_ | Concise, actionable guidelines | - transparent indications of symptoms - clear, quantifiable exercise limitations - tailored exercise plans | *“It was me guessing what I could do. And then asking ‘is it OK if I run?’ [...] The answer would always be ‘yeah. If you feel like you can do that, you should do that’. [...] That's not very precise because it is a heart condition, right? So you don't want to overdo it.”* (P7) |
| V_6_ | Trustworthy, readily available support | - punctual follow-ups and feedback - collaboration when making health-related decisions - streamline communication - complete trust in clinicians | *“I think that the communication with the cardiologist is a bit slow - the E-consult. Every time you send a message, somebody else has to check it. […]  I put it through and you have to wait 2-3 days [...] and then he says ‘Do this and come back to me in a week’, but they only keep the e-consult open for six days” (P3)* |
| V_7_ | Care with 'a personal touch' | - human interaction - emotional support (from clinicians) - reassurance, encouragement and feedback (from clinicians) | *“You are put on a bike, and then they come with the heart rate monitor. They’re all busy, I know that. […] They don't have to talk for an hour, but a short go-around would be nice.”* (P6) |
| V_8_ | In and outside hospital oversight | - pre-program supervision and guidance - feel looked after by a healthcare professional/ ‘be part of something’ - feeling reassured and calm through supervision | *“I would start in the hospital [guidance[, that would give me certainty that I am doing well. Then that could be phased out, so that at a certain point you only do it at home.”* (P15) |
| V_9_ | Emotional support and sharing | - being around people with the same condition - sharing experiences with family and close ones | *“I think very important [i.e., the wife’s role in CR]. She knows exactly what happened to me. She knows the status of my heart and body, and how it has recovered.“* (P14) |
| *Data and technology-centric* | | | |
| V_10_ | Health and performance quantification | - progress and performance measurements - routine measurements to check in with one's body - self-monitoring for staying  within exercise limitations | *“It is important to measure where you are now. […] Blood pressure, heart rate. [...] That gives confidence. Confidence in your own body gives you another chance to take the next step.”* (P5) |
| V_11_ | Clinical validation on information and data | - checking sensor data with clinicians - discussing implications of data with clinicians - receiving clinically-validated information | *“ [..] I already googled about sports [in CR]. But then you get a bunch of different websites saying different things.”* (P3) |
| V_12_ | Reliable information systems | - accurate sensors and data - easy-to-use, understandable systems - robust and simple infrastructure - technical support | *“I think that quite a lot of people now have a smartwatch. Especially athletes. And they can use their own smartwatch. I think that is really an added value. Not that they get another one and they don't know how it works.”* (HCP2 |
