# Supplementary material for: Design Requirements for Cardiac Telerehabilitation Technologies Supporting Athlete Values: Qualitative Interview Study
Source: JMIR Rehabil Assist Technol. 2025 Apr 17;12:e62986. doi: 10.2196/62986 (PMC12046260; doi:10.2196/62986)
Supplement: Multimedia Appendix 5 [file rehab_v12i1e62986_app5.pdf]

Card-sorting activity results

Box 1 - Legend

Focus group (FG) results - cards sorted from most to least preferred. FG 5 and 6 were composed of healthcare professionals (HCPs).

++ Elements characterized as *must have*.

+ Elements characterized as *nice to have*.

- Elements characterized as *not needed*.

* Participants could not agree on their preference for this card.

Each feature is color-coded and marked with the corresponding symbol according to the category it belongs to: ▲*Education and assistance*, ■*Consultations, coaching, and guidance*, ★*Monitoring behaviors for supervision and oneself*, and ♦*Co-experience (social aspect)*.

CTR feature	FG1	FG2	FG3	FG4	FG5 (HCPs)	FG6 (HCPs)
■Having periodic checks/consultations (e.g. with a clinician, periodic surveys, or with a virtual agent)	++	++	++	++	++	++
■Receiving feedback from clinicians when there is a red flag in my data (e.g., heart rate too high for too long)	++	++	++	++	++	++
▲Technical assistance and instructions on how to use the technology, share the data etc.	++	++	++	++	++	++
■Personalized clinical recommendations and planned activities (e.g., training schemes and schedules, limitations for how much I can do)	++	++/+*	++	++	++	++

CTR feature	FG1	FG2	FG3	FG4	FG5 (HCPs)	FG6 (HCPs)
★Using sensors to monitor my activities and behaviors (e.g., mobile phone, wearable - watch, breast belt etc.)	++	++	++	++	+	++
★Collaboratively adapting my goals together with my clinician based on my personal situation	++	+	++	++	++	++
■The system notifies clinicians when there are red flags in my data (e.g., heart rate is too high for a long time, or performance is above limitations)	++	++	++/+*	++	+	++
▲Using the technology I already own for self-monitoring	++	++/+*	++	++	++	+
■Having in-person consultations with a clinician	-	++	++	++	++	++
■Training in a group (e.g., through video conferencing or face-to-face), doing standardized cardiac rehabilitation exercises, with live or remote supervision	++	+/-*	++	++	++	++/+*
■Clinicians can receive and see all the data collected by the system	++	++	++	++	-	++
★Receiving notifications when there are red flags in my data (eg., heart rate is too high for a long time, or performance is above limitations)	++	+	+	++	++	++
■Training independently at home (doing my own sports), with periodic checks on my workouts through remote supervision	++	++/+*	++	++	+	+
■Request on-demand consultations whenever I need to (e.g. with a clinician, through a survey, or with a virtual agent)	++	+	+	++	++	+
■Sharing representations of my data during consultations to my clinician (e.g., to discuss it or ask questions)	+	++	++	++	+	+
■Receiving support from clinicians regarding my emotions and worries	-	+	++	++	++	++

CTR feature	FG1	FG2	FG3	FG4	FG5 (HCPs)	FG6 (HCPs)
★Seeing performance and health data in a graphic form - e.g., graphs, animations, icons etc.	++/+*	+	+	++	++/+*	++
▲Having access to educational materials in digital form (e.g., videos and tutorials, information web pages)	+	++	++	+	+	++
▲Being able to take part in educational group sessions moderated by a clinician (e.g., online or face to face)	+	++	++	++	+	+
▲Being able to search for clinically validated information anytime I need to (e.g., about my condition, medication, fears)	++	+	++	++	+	+
■Being able to provide feedback to clinicians about the training sessions - e.g., how I felt during the training and how it can be improved	-	+	++	++	++	+
★Setting short and long-term goals for myself - e.g., how much sports I do per week, at which intensity	+	+	++	+	++	+
★Seeing comparisons between performed workouts versus my clinical recommendations - e.g., suggested max heart rate next to your actual heart rate	+	+	+	+	++	++
★Seeing comparisons between performed workouts versus my clinical recommendations - e.g., suggested max heart rate next to your actual heart rate	+	+	+	+	++	++
▲Having the hospital provide me with the technology I need (e.g., a wearable sensor and a tablet/mobile phone)	+	+	++	++	+	+
■Receiving feedback on my performance and health data during my workouts	++	+/-*	+	++	+	+
★Receiving personalized training goals from my clinician (e.g., goals based on my previous athletic performance)	-	++	++	+	-	++

CTR feature	FG1	FG2	FG3	FG4	FG5 (HCPs)	FG6 (HCPs)
▲Instructions about why it is important to monitor myself and how it can benefit my health	-	-	+	++	++	++
★Periodical reminders if data has not been entered for a while	+	-	+	+	+	++
♦Receiving peer-based support from other athletes with cardiac problems - e.g., on my training or my trajectory	++	-	+	+	+	+
▲Being able to personalize the technology (e.g., making my own avatar or setting reminders and feedback based on my own preferences)	-	+	-	++	++	+
★Reporting on subjective experiences through diaries or surveys - e.g., emotions, worries, side effects, stress, social interactions etc.	+	-	-	++	-	++/+*
♦Connecting and communicating with other patients through an online community (e.g., for communication, sharing experiences, cooperation)	-	-	++	+/-*	+	+
■I am able to choose myself which data my clinician is able to see (e.g., only showing activity and sleep, hiding stress)	-	-	-	++/+*	+	+
■Receiving periodical feedback and recommendations from a virtual assistant (i.e., an automated system) on my current behaviors and health data	+	+/-*	-	-	+	+
★Periodical reminders about targets or habits I need to work on	-	-	-	+	+	+/-*
■I am able to manually send my data to clinicians whenever I want to	+	-	-	+	-	+
★Having periodical motivational messages on 'bad days' or when almost reaching a goal	-	+/-*	-	-	+	++/+*

CTR feature	FG1	FG2	FG3	FG4	FG5 (HCPs)	FG6 (HCPs)
♦Sharing my data with my family and/or friends (e.g., for discussions, sharing concerns, making decisions together, planning activities)	-	++	-	-	-	+
♦Having means or channels that allow me to train with other peers (e.g., other athletes with cardiac problems)	-	-	+	-	+	+
★Rewards like points or trophies when achieving certain goals or targets	+	+/-*	-	-	-	+
♦Sharing red flags in my data (e.g., heart rate is too high for a long time, or performance is above limitations) with my family or friends	-	++/-*	-	-	-	-
■Being able to include my family/friends to participate in trainings with me	-	-	-	-	+	-
▲Asking for information from a virtual assistant (e.g., automated chatbot)	-	-	-	+/-*	-	+/-*

Table 1. Results of the card-sorting focus group. Legend in Box 1.
