# Supplementary material for: Design Requirements for Cardiac Telerehabilitation Technologies Supporting Athlete Values: Qualitative Interview Study
Source: JMIR Rehabil Assist Technol. 2025 Apr 17;12:e62986. doi: 10.2196/62986 (PMC12046260; doi:10.2196/62986)
Supplement: Multimedia Appendix 6 [file rehab_v12i1e62986_app6.docx]

Derived CTR technology requirements paired with athlete values, where `**x**` indicates a match between a feature and a value, cards used to derive the requirements and example notes or observations made during the card-sorting activity

| **Technology requirements** | **V_1_** | **V_2_** | **V_3_** | **V_4_** | **V_5_** | **V_6_** | **V_7_** | **V_8_** | **V_9_** | **V_10_** | **V**_11_ | **V_12_** | **Related cards** | **Example FG notes** |
| --- | --- | --- | --- | --- | --- | --- | --- | --- | --- | --- | --- | --- | --- | --- |
| *Supporting remote monitoring* | | | | | | | | | | | | | | |
| Remotely monitored sport-specific training (e.g., type of sport, duration, frequency, HR (zones), degree of effort) and biophysical measurements (e.g., blood pressure) - before, during, CR program | **x** |  |  |  |  |  |  | **x** |  |  |  |  | Training independently at home (own sports), with remote supervision | *Start training at home with supervision – esp. after hospitalization (FG3)*  *Impossible to monitor at home without technology (FG2)* |
|  |  |  |  |  |  |  |  |  |  |  |  |  | Monitoring activities and behaviours through sensors |  |
| Exercise data collection and aggregation from athlete’s own wearable sensors (e.g., sports watches, chest straps, mobile applications)a; the hospital provides patients with sensors in case they do not use one |  |  |  |  |  |  |  | **x** |  | **x** |  | **x** | Using the technology I already own for self-monitoring | *Sporters who have their own technology can use it; if they do not, then the hospital should provide it  (FG1)* |
|  |  |  |  |  |  |  |  |  |  |  |  |  | Having the hospital provide me with the technology I need (e.g., a wearable sensor and a tablet/mobile phone) |  |
| Simple, brief, open approach to subjective data collection gathering contextual information (e.g.,  symptom uncertainty, sport-related anxiety, questions about sensor data irregularities) |  |  |  |  |  |  | **x** | **x** | **x** |  |  |  | Reporting on subjective experiences through diaries or surveys - e.g., emotions, worries, side effects, stress, social interactions etc. | *Not if long questionnaires are needed (FG2,4)*  *Emotional support is very important (FG3)*  *Look at the person as a whole and not only at their heart problem (FG5)*  *Useful as explanation for data (FG6)* |
|  |  |  |  |  |  |  |  |  |  |  |  |  | Receiving clinician support about emotions and worries |  |
| Technical instructions and online assistance in monitoring, syncing, sharing, and correctly interpreting the data (e.g., instructional videos or written information) |  |  |  |  |  |  |  |  |  |  |  | **x** | Technical assistance and instructions on how to use the technology, share the data etc | *If there is a connection between commercial applications (e.g., Strava/Polar) then that needs to be explained (FG1)*  *Necessary when “getting stuck” or for less technical users (FG2)* |
|  |  |  |  |  |  |  |  |  |  |  |  |  | Instructions about why it is important to monitor myself and how it can benefit my health |  |
| Continuous and automatic transmission of remote data to clinicians; opting for manual, discrete transmission is possible |  |  |  |  |  | **x** |  | **x** |  |  | **x** | **x** | Clinicians can receive and see all the data collected by the system | *Does not matter if data is sent automatically or manually, as long as any professional looks at the data (FG1) Do not mind sharing all the data, but it is nice if able to make a decision whether to share all the data or not (FG4)* |
|  |  |  |  |  |  |  |  |  |  |  |  |  | The system automatically sends my data to my clinicians (e.g., in real time, or periodically) |  |
|  |  |  |  |  |  |  |  |  |  |  |  |  | I am able to manually send my data to clinicians whenever I want to |  |
| *Supporting human-data interaction* | | | | | | | | | | | | | | |
| Graphs displaying training progress (e.g., HR, intensity minutes) and sessions (e.g., duration, HR zones), contrasting with limitations and recommendations (e.g., exceeding max HR), for easy understanding by trainers, family, and clinicians |  |  | **x** |  | **x** |  |  |  |  | **x** |  |  | Seeing performance and health data in a graphic form - e.g., graphs, animations, icons etc. | *Rehabilitation is about progress - graphs are valuable to demonstrate the progress* (FG1)  *Makes the information clear and insightful* (FG4) |
|  |  |  |  |  |  |  |  |  |  |  |  |  | Seeing comparisons between performed workouts versus my clinical recommendations |  |
| Instant alerts for exceeding clinical limitations during training (e.g., excessive time spent in HR zone 4) |  |  | **x** |  |  | **x** |  |  |  | **x** |  |  | Receiving notifications when there are red flags in my data (eg., heart rate is too high for a long time, or performance is above limitations) | *It feels safe to receive notifications about red flags*  (FG1)  *If the heart rate gets dangerously high during a workout, it is nice to receive a notification* (FG2) |
|  |  |  |  |  |  |  |  |  |  |  |  |  | Receiving feedback on my performance and health data during my workouts |  |
| Alerts for prolonged anomalies in data (e.g., exceeding limitations, signs of cardiac arrhythmia), with follow-up recommendations for action – either adjusting performance or seeking clinical assistance |  | **x** | **x** |  | **x** |  |  |  |  | **x** |  |  | The system notifies clinicians when there are red flags in my data (e.g., heart rate is too high for a long time, or performance is above limitations) | *Professionals should know if it is  not going well* (FG4)  *It is useful when the professional receives a notification - but the system should keep the responsibility with the patient* (FG5) |
|  |  |  |  |  |  |  |  |  |  |  |  |  | Receiving feedback from clinicians when there is a red flag in my data (e.g., heart rate too high for too long) |  |
| Selecting and sharing monitored data snippets (e.g., HR from a specific exercise session) with clinicians within the system, facilitating remote data-focused discussions |  |  |  |  |  |  |  |  |  |  | **x** |  | Sharing representations of my data during consultations to my clinician (e.g., to discuss it or ask questions) | *Nice way for HCPs to get insights in the data, as well as for “passionate athletes” who keep track of their data. Collaboratively working on health goals and next steps is a must.* (FG5) |
|  |  |  |  |  |  |  |  |  |  |  |  |  | Receiving personalized training goals from my clinician (e.g., goals based on my previous athletic performance) |  |
| An overview of clinical test results such as exercise stress tests and clinical scans |  |  | **x** |  |  |  |  |  |  | **x** |  |  | Access to personal clinical details such as scans and tests made by the doctor *  *empty card feature added by FG1 | *Access to personal clinical details such as scans and tests made by the doctor* (FG1)  *Connect collected data  to program evaluations to observe progress* (FG6) |
| Sharing health and exercise data with family, including exercise location and alarming signals, for discussion, negotiation, and easing concerns |  |  | **x** |  |  |  |  |  | **x** |  |  |  | Sharing my data with my family and/or friends (e.g., for discussions, sharing concerns, making decisions together, planning activities) | *Beneficial for patients living alone, whose family wants to know how they are from a distance* (FG2) |
|  |  |  |  |  |  |  |  |  |  |  |  |  | Sharing red flags in my data (e.g., heart rate is too high for a long time, or performance is above limitations) with my family or friends |  |
| *Supporting remote coaching* | | | | | | | | | | | | | | |
| Setting, storing, and reviewing personal health and sport-related goals; sharing them with clinicians for feasibility assessment and feedback and personalization | **x** | **x** |  | **x** |  |  |  | **x** |  |  | **x** |  | Collaboratively adapting my goals together with my clinician based on my personal situation | *Preferably they do not want to receive them, but set their own goals and hear from a professional whether it is realistic; important to collaboratively set goals in the first 3 months* (FG1) *Setting goals motivates them to find out if they can do better every time* (FG3) |
|  |  |  |  |  |  |  |  |  |  |  |  |  | Setting short and long-term goals for myself - e.g., how much sports I do per week, at which intensity |  |
|  |  |  |  |  |  |  |  |  |  |  |  |  | Receiving personalized training goals from my clinician (e.g., goals based on my previous athletic performance) |  |
| Tailored content including clinically validated, personalized training schemes tailored to one’s sport, goals, current condition, and past performances | **x** |  | **x** | **x** | **x** |  |  |  |  |  | **x** |  | Personalized clinical recommendations and planned activities (e.g., training schemes and schedules, limitations for how much I can do) | *Trainings personalized to the capabilities of the participant, including post-CR* (FG1)  *The first 3 months, personalized training schemes* (FG2)  *Want to know where personal limits are* (FG3) |
| Annotating clinical training suggestions with feedback grounded in personal experiences |  | **x** |  |  |  |  | **x** |  |  |  |  |  | Being able to provide feedback to clinicians about the training sessions - e.g., how I felt during the training and how it can be improved | *A must have as this is the essence of the CR training* (FG5)  *Patients feel the need to reflect on their training, if it was too heavy for example* (FG6) |
| Receiving clinical feedback on current performance (e.g., through annotations on remotely monitored exercise data) | **x** |  |  |  |  | **x** |  |  |  |  | **x** |  | Receiving support from clinicians regarding my emotions and worries | *Important to know what they are doing well and what not, especially in the beginning when there is a lot of insecurity* (FG3) |
|  |  |  |  |  |  |  |  |  |  |  |  |  | Receiving feedback on my performance and health data during my workouts |  |
| Multi-media, digital information resources about athletic patients with heart problems and how to deal with exercising while having a cardiac condition |  |  | **x** |  |  |  |  |  |  |  | **x** |  | Having access to educational materials in digital form (e.g., videos and tutorials, information web pages) | *Replacing brochures with digital material (brochures are “a bit passée” and repetitive)* (FG2) *Internet information is scattered and unreliable - the need for information specifically for sporters with heart problems* (FG1) |
|  |  |  |  |  |  |  |  |  |  |  |  |  | Being able to search for clinically validated information anytime I need to (e.g., about my condition, medication, fears) |  |
| *Supporting remote consultations* | | | | | | | | | | | | | | |
| Periodic clinical check-ins on exercise management (e.g. updating knowledge on the exercise limits or exercise-related symptoms) through synchronous or asynchronous channels such as video calls, or online surveys |  |  | **x** |  |  | **x** |  | **x** |  |  | **x** |  | Having periodic checks/consultations (e.g. with a clinician, periodic surveys, or with a virtual agent) | *The need to be able to ask what is allowed and what is not allowed; preferably an e-consult for short questions* (FG1) *Especially in the beginning of CR there should be face-to-face contact* (FG3) |
|  |  |  |  |  |  |  |  |  |  |  |  |  | Having remote consultations (e.g., by phone, through texting or video calling) |  |
| E-consult functionality allowing users to ask specific questions to appropriate clinicians via chat functionalities |  |  |  |  |  | **x** | **x** | **x** |  |  |  |  | Request on-demand consultations whenever I need to (e.g. with a clinician, through a survey, or with a virtual agent) | *Being able to contact a professional when questions arise; could be a message/ e-mail/etc.* (FG4) |

LEGEND:
V1 - A dynamic lifestyle
V2 - Independence and confidence in one's body
V3 - Coming to terms with one's condition
V4 - A goal and performance-oriented approach
V5 - Concise, actionable guidelines
V6 - Trustworthy, readily available support
V7 - Care with 'a personal touch'
V8 - In and outside hospital oversight
V9 - Emotional support and sharing
V10 - Health and performance quantification
V11 - Clinical validation on information and data
V12 - Reliable information systems
